# Machine Learning-Enhanced Calculation of Quantum-Classical Binding Free Energies

**DOI:** 10.1021/acs.jctc.5c00388

**Published:** 2025-08-05

**Authors:** Moritz Bensberg, Marco Eckhoff, F. Emil Thomasen, William Bro-Jørgensen, Matthew S. Teynor, Valentina Sora, Thomas Weymuth, Raphael T. Husistein, Frederik E. Knudsen, Anders Krogh, Kresten Lindorff-Larsen, Markus Reiher, Gemma C. Solomon

**Affiliations:** † Department of Chemistry and Applied Biosciences, ETH Zurich, Vladimir-Prelog-Weg 2, Zurich 8093, Switzerland; ‡ Department of Biology, Linderstrøm-Lang Centre for Protein Science, 4321 University of Copenhagen, Ole Maaløes Vej 5, Copenhagen N DK-2200, Denmark; § Department of Chemistry and Nano-Science Center, University of Copenhagen, Universitetsparken 5, Copenhagen Ø DK-2100, Denmark; ∥ NNF Quantum Computing Programme, Niels Bohr Institute, University of Copenhagen, Blegdamsvej 21, Copenhagen Ø DK-2100, Denmark; ⊥ Department of Computer Science, University of Copenhagen, Universitetsparken 1, Copenhagen Ø DK-2100, Denmark; # Department of Computer Science, and Center for Health Data Science, Department of Public Health, University of Copenhagen, Universitetsparken 1, Copenhagen Ø DK-2100, Denmark

## Abstract

Binding free energies are key elements in understanding and predicting the strength of protein–drug interactions. While classical free energy simulations yield good results for many purely organic ligands, drugs, including transition metal atoms, often require quantum chemical methods for an accurate description. We propose a general and automated workflow that samples the potential energy surface with hybrid quantum mechanics/molecular mechanics (QM/MM) calculations and trains a machine learning (ML) potential on the QM/MM energies and forces to enable efficient alchemical free energy simulations. To represent systems including many different chemical elements efficiently and to account for the different descriptions of QM and MM atoms, we propose an extension of element-embracing atom-centered symmetry functions for QM/MM data as an ML descriptor. The ML potential approach takes electrostatic embedding and long-range electrostatics into account. We demonstrate the applicability of the workflow on the well-studied protein–ligand complex of myeloid cell leukemia 1 and the inhibitor 19G and on the anticancer drug NKP1339 acting on the glucose-regulated protein 78.

## Graphical abstract

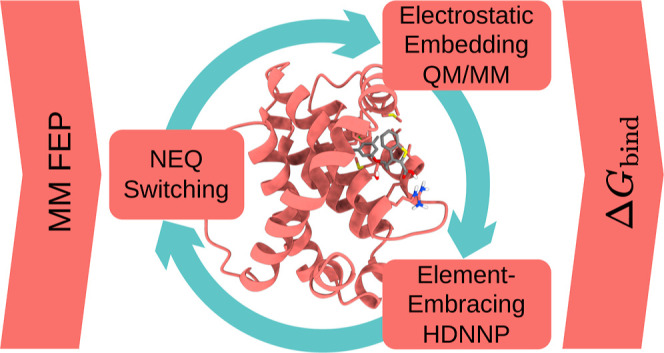

## 1 Introduction

Molecular interactions such as protein–protein, protein–DNA, and protein–RNA interactions are essential for biological functions.^1^ For example, abnormal protein–protein interactions can cause cancer and neurodegenerative diseases.^2^ Molecular interactions also determine whether a drug molecule will bind to a protein target. A key quantity in this binding process is the free energy. If a reduction in free energy is accessible and larger than that in competing processes, the drug molecule will preferentially bind to the target protein. This process is called molecular recognition. While there are many molecular mechanisms underlying how drugs assert their functions, such as activating, inhibiting, or targeting a protein for degradation, an essential aspect is binding with sufficient affinity and specificity. Therefore, predicting binding free energies with high accuracy through computational models would allow for targeted and rapid design of new drugs with reduced side effects, reduced risk that bacteria and viruses develop resistance, and reduced economic costs.^3–5^ Furthermore, accurate predictive models for molecular interactions can support improved enzyme engineering for technological applications and, more generally, improve understanding of fundamental biological processes.^6^

With these considerations in mind, there is clear utility in a generally applicable computational workflow to determine binding free energies from first principles for arbitrary and complex biomolecular systems. Such a workflow should be automated to reduce human time investment and economic cost.^7^ While finite computational resources necessitate the introduction of various approximations, the goal is an approach that is systematically improvable with increasing computing power.

Molecular dynamics (MD) simulations can be employed to determine the protein–ligand binding free energies. In the simplest approach, the binding free energy can be calculated directly from a standard MD simulation employing the equilibrium populations of the bound and unbound states. However, it can be a major challenge to sample the binding–unbinding process sufficiently. Several different approaches have been developed to solve this sampling problem. One of the most powerful methods is alchemical free energy (AFE) simulations. It bridges the thermodynamic states of interest, e.g., the bound and unbound states, with unphysical (alchemical) intermediate states of the system, circumventing sampling of the binding/unbinding process.^8–10^ Alternatively, a bias potential can be added to overcome the energetic barrier separating the bound and unbound states, such as in metadynamics and umbrella sampling.^11–15^

Previous studies have shown that free energy calculations with classical force fields can efficiently represent biomolecular systems in solution and may approach experimental accuracy of binding free energies.^16,17^ However, the applicability of a classical force field is often limited to drug molecules containing main-group elements and those for which experimental data aid in the parametrization of the force field. These limitations can lead to excellent drug candidates being missed in drug discovery attempts, such as platinum-based drugs, which are well-known chemotherapeutic agents.^18^ Unfortunately, parameterizing classical force fields for new elements is far from trivial. Moreover, they are challenging to improve systematically.^19^

In contrast to classical force fields, quantum mechanical electronic structure methods can be employed for any element. They typically yield more accurate energies and forces due to their explicit treatment of electrons. However, the increase in accuracy and flexibility also results in a significantly higher computational cost. This cost prohibits large-scale sampling of the conformational space of complex molecular systems, which is necessary to accurately estimate the entropic contribution to the free energy. Extensive sampling is especially relevant for biomolecules due to their flexibility, which leads to a large conformational space accessible at physiological temperatures.^20,21^

Embedding methods are a powerful tool to circumvent the limitations of both classical force fields and quantum mechanical electronic structure methods. In a hybrid quantum mechanics/molecular mechanics (QM/MM) approach,^22,23^ a small QM region is described by a quantum mechanical electronic structure method, while the remaining atoms are represented by force fields. In this way, the QM/MM approach combines an accurate representation of the region of interest, e.g., a catalytic center in an enzyme or a drug molecule in a protein’s binding pocket, with an efficient description of the remaining protein and solvent. We note that another approach to incorporate QM/MM information in a sample-efficient manner is the reformulation of the force field expression to include higher-order physical effects such as multipolar electrostatics and anisotropic polarization.^24^

Even with the advantages of QM/MM approaches, sampling driven by QM/MM methods will be demanding if the relevant region for the binding process is large, a computationally costly electronic structure method is required, or the sampling procedure converges slowly. Furthermore, AFE approaches employ unphysical intermediate states that cannot easily be described with traditional QM/MM methods. Therefore, QM/MM has previously been applied as an additional correction to an initial binding free energy calculated with MM through QM/MM energy evaluation on MM structures.^25,26^ These approaches avoid explicit propagation on the QM/MM potential energy surface (PES). However, they implicitly assume that the MM structures are also sufficiently representative of the structures that would occur in a QM/MM simulation, which may be qualitatively incorrect.^27^ Explicit propagation on the QM/MM^28^ or mixed MM and QM/MM PESs, e.g., through nonequilibrium switching,^29,30^ can remedy this problem at the high computational cost of QM/MM. Alternatively, intermediate reference potentials that are parameterized with the target QM method help bridge the gap between the MM and QM/MM PES.^28^

To increase the sampling efficiency, machine learning (ML) potentials^31–34^ can be applied to enable explicit propagation on the QM/MM PES. ML potentials are able to retain the high accuracy of the PES obtained by a quantum mechanical electronic structure method, while their computational efficiency is multiple orders of magnitude higher,^35^ reaching simulation times of nanoseconds per day.^36^ Despite their promising performance for a wide variety of problems and systems,^37–40^ the application of ML potentials in the context of protein–drug interactions is still challenging.^41–44^ This challenge persists despite the development of software frameworks that facilitate the use of ML potentials trained to reproduce the QM/MM PES.^45–47^ In hybrid QM/MM approaches, the interactions among QM atoms, among MM atoms, and between QM and MM atoms are treated differently. However, standard ML potentials normally do not distinguish between these interaction types. Furthermore, many structural descriptors applied as features for standard ML potentials cannot deal efficiently with a large number of different chemical elements occurring in protein–drug complexes. Therefore, many previous studies on ML potentials for AFE simulations were limited by the restricted applicability of pretrained ML potentials, treated the interactions between QM and MM atoms only on the MM level using fixed point charges,^48–50^ predicted the charges on the fly during the simulation,^50–52^ or included the electrostatic potential of the MM atoms in the ML descriptor.^53^

As an alternative, Δ-ML strategies^54^ can be employed, which learn a correction on top of energies and forces calculated by a low-cost quantum chemical method, typically a semiempirical QM method. Because the low-cost quantum chemical method already provides a qualitative description of the QM atoms, including the interaction between QM and MM atoms, the complexity of the data that the ML potential must learn is reduced, as demonstrated in the context of QM/MM in refs 33 and 55 . However, evaluating the low-cost quantum chemical method is typically slower, or at least as slow as, evaluating the ML potential. Therefore, the computational cost of Δ-learning approaches increases significantly.

Recent work has shown that, for example, a high-dimensional neural network potential (HDNNP)^31,32,56^ is able to represent a QM/MM PES including polarization of the QM atoms by MM point charges.^33^ However, this approach employs modified atom-centered symmetry functions (ACSFs) as features, which show unfavorable scaling with the number of different elements. One approach to mitigate this unfavorable scaling is applying element-embracing atom-centered symmetry functions (eeACSFs).^57^ In this work, we combine these two methods by proposing an extension of eeACSFs, which makes them applicable to QM/MM data. In this way, we can incorporate accurate electronic structure energies obtained in representative chemical environments through efficient ML potentials into large-scale AFE simulations of binding free energies.

In addition to an increase in accuracy, the approach should be integrated into an automated pipeline to decrease human time investment. Therefore, we propose an end-to-end pipeline utilizing distributed computing that begins with system preparation and ends with binding free energy prediction.

Computationally demanding tasks such as QM/MM calculations can be distributed to multiple computing centers with one central database coordinating the prioritization of tasks and storing results by exploiting the SCINE framework.^58^ Additional details on the computational framework are provided in the Supporting Information. We apply active learning by a query-by-committee strategy^59–62^ to complete the sampling of QM/MM training data in a self-driven way. We then perform AFE simulations and nonequilibrium (NEQ) switching simulations to correct the end state of classical MM simulations.^63^ These end-state corrections are driven by the hybrid ML/MM approach, which samples the targeted conformational space directly.

## 2 Protein–Ligand Complexes

We chose two protein–ligand complexes to test our approach. The first system is myeloid cell leukemia 1 (MCL1) protein bound to the small molecule 19G^64^ (see Figure 1). MCL1 is a modulator of apoptosis in the BCL2 protein family. Dysregulation of MCL1 is associated with various cancers, and several small-molecule inhibitors for MCL1 have been designed for cancer therapy.^65^ MCL1–19G is a suitable model system for our purpose for several reasons: (i) MCL1 is a small protein (150 residues), (ii) the structure of the complex has been solved by X-ray crystallography,^64^ (iii) the MCL1–19G affinity has been measured experimentally,^64^ and (iv) relative AFE calculations have previously been performed for MCL1–19G binding.^17,66^

**Figure 1:**
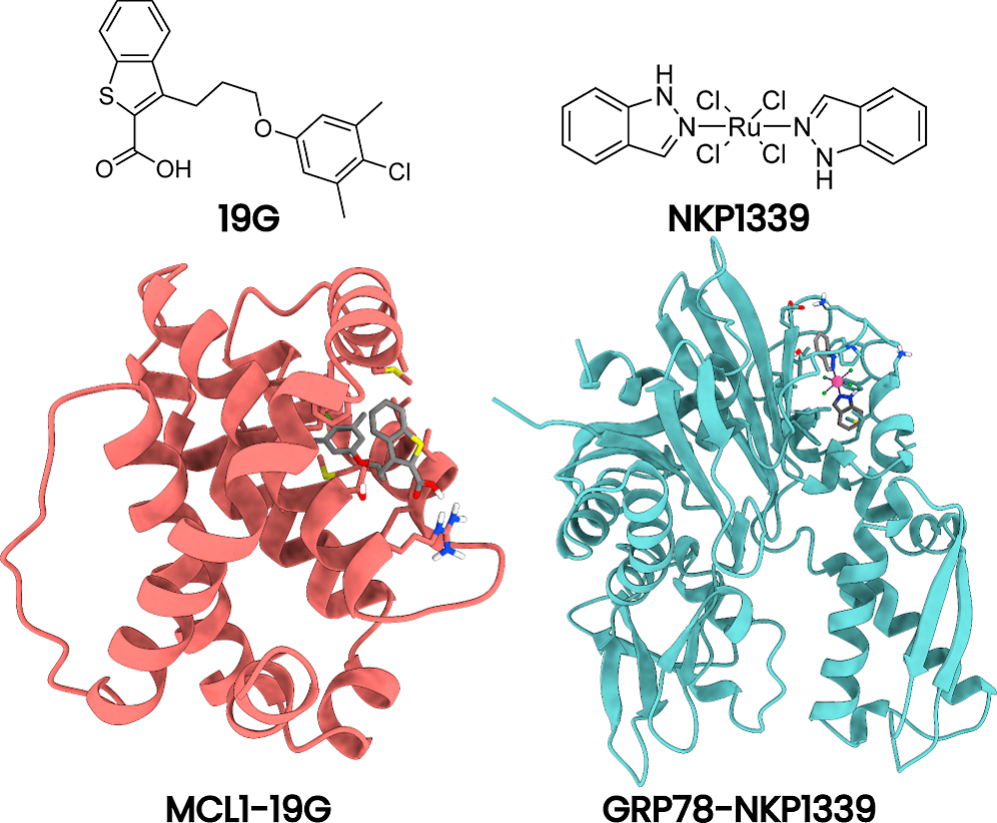
Illustration of the two investigated systems MCL1–19G (left) and GRP78–NKP1339 (right). The ligands are at the top, and the protein–ligand complexes are at the bottom.

The second protein–ligand system is the ATPase domain (residue 24-407) of the 78 kDa glucose-regulated protein (GRP78) bound to the small molecule NKP1339. GRP78 is an HSP70 chaperone in the endoplasmic reticulum (ER), where it facilitates protein folding, degradation, and translocation across the ER membrane, as well as regulation of the unfolded protein response associated with ER stress.^67^ GRP78 also functions as a receptor on the cell surface.^68^ Elevated GRP78 levels and increased localization to the cell membrane are associated with a variety of cancers and the development of chemoresistance.^69,70^ NKP1339 is a small-molecule anticancer compound that targets GRP78.^71,72^ Phase I clinical trials have shown that NKP1339 has modest antitumor activity and a manageable safety profile.^73^ The GRP78–NKP1339 system is a suitable test for our hybrid QM/MM approach because NKP1339 contains a central ruthenium atom, which is typically not parameterized in MM force fields.

## 3 Methods

### 3.1 Thermodynamic Cycle

AFE simulations are routinely performed with MM models. These models are ideal as different interactions can be independently tuned; the Lennard-Jones and Coulomb interactions between molecules can gradually be turned off through a series of states defined by a scaling parameter λ, which scales the nonbonded interactions between specified parts of the system. For example, the ligand can be decoupled from its environment through a series of simulations interpolating between λ = 1, where the system is fully interacting, and λ = 0, where the ligand is in a vacuum with the remainder of the system still interacting.

To estimate the free energy differences between the series of states, we utilize the Multistate Bennett acceptance ratio (MBAR) free energy estimator.^74^ MBAR takes into account the energies of the configurations sampled at every thermodynamic state evaluated with the energy function of each thermodynamic state. This estimation is done by self-consistently solving a system of equations defining the free energy at each thermodynamic state

1 $$f_{i}=-\ln\sum_{j=1}^{K}\sum_{n=1}^{N_{j}}\frac{\exp\left[-u_{i}\left(x_{jn}\right)\right]}{\sum_{k=1}^{K}N_{k}\exp\left[f_{k}-u_{k}\left(x_{jn}\right)\right]}$$

where *f*_*i*_ is the free energy of thermodynamic state *i*, *K* is the total number of thermodynamic states, *N* is the number of configurations, *u*(*x*) is the energy of the configuration *x* evaluated with the energy function *u*, and *f*_*k*_ is the free energy of another thermodynamic state *k*. The gradual decoupling of interactions using a series of λ values is carried out to ensure that there is sufficient overlap among the energy distributions of neighboring states. This approach leads to an accurate estimate of the free energy differences with MBAR. The overlap can be further improved by applying a Hamiltonian replica exchange scheme, which allows simulations of different thermodynamic states to swap their energy functions on the fly.^9,75^

An advantage of MM models for AFE simulations is that they are numerically robust, even for unphysical states. These states are encountered if the interaction strength is very low, which can lead to extremely short interatomic distances. To describe these states with ML potentials, the ML potential has to be specifically designed for that purpose.^76^ To avoid this issue, we employ an end-state correction strategy,^29^ exploiting that the free energy is a state function. This fact allows us to simulate the alchemical path with the MM force field and then formally switch the PES from MM to ML/MM, correcting for any differences in the PESs, as demonstrated, for instance, for the ANI-2x^78^ ML potentials using mechanical embedding^63,77^ and a Δ-ML strategy by York and co-workers, including electrostatic embedding.^55^ Note that electrostatic embedding was found to be crucial for systematically improving on classical MM force fields.^79^

In AFE simulations, the ML/MM binding free energy of a ligand to a protein Δ*G*_bind_^ML/MM^ is indirectly calculated as illustrated in the thermodynamic cycle in Figure 2. Initially, the change in free energy between the solvated protein–ligand complex and the solvated protein with the gas phase ligand is determined in AFE simulations employing an MM force field (Δ*G*_complex_^MM^ in Figure 2). Subsequently, the change in free energy between the solvated ligand and the gas-phase ligand is calculated (Δ*G*_solv_^MM^ in Figure 2). The change in free energy between the two states with the ligand in the gas phase is zero (Δ*G* = 0). Therefore, the free energy of binding can be determined as

2 $$\Delta G_{bind}^{MM}=\Delta G_{complex}^{MM}-\Delta G_{solv}^{MM}$$

**Figure 2:**
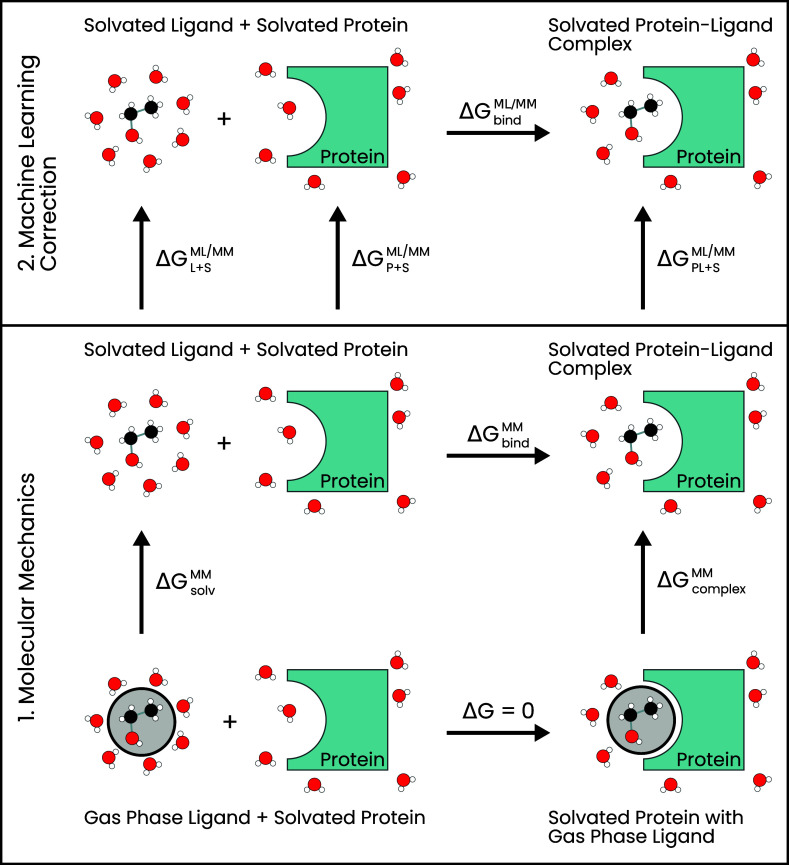
Thermodynamic cycle for calculating the binding free energy with AFE simulations employing MM (bottom box) and ML/MM end-state corrections (top box). The gray-shaded circles represent that the interactions between the ligand and the protein and the solvent have been scaled to zero. The white spheres are hydrogen, the red spheres are oxygen, and the black spheres are carbon.

Next, we calculate the correction to the free energy associated with going from the MM description to the ML/MM description for the end states in the thermodynamic cycle (top box of Figure 2). We apply NEQ switching simulations to determine the free energy difference between MM and ML/MM for protein–ligand complex Δ*G*_PL+S_^ML/MM^ and for the ligand in solution Δ*G*_L+S_^ML/MM^.

There is no need to calculate an ML/MM correction for the gas-phase ligand, as this correction does not contribute to the thermodynamic cycle. Furthermore, the ML/MM correction for the solvent Δ*G*_S_^ML/MM^ and the protein in solution Δ*G*_P+S_^ML/MM^ are zero in our approach because we restrict the ML potential to the ligand. This restriction is reasonable since the protein and solvent can be described by specialized and accurate MM force fields, which are not available for arbitrary ligands.

Adding all corrections to eq 2, we obtain

3 $$\Delta G_{bind}^{ML/MM}=\Delta G_{complex}^{MM}+\Delta G_{PL+S}^{ML/MM}-\Delta G_{solv}^{MM}-\Delta G_{L+S}^{ML/MM}$$

### 3.2 Workflow

As established in Section 3.1, we need to calculate Δ*G*_complex_^MM^ and Δ*G*_solv_^MM^ with MM-driven AFE simulations, as well as the end-state correction terms for the protein–ligand complex Δ*G*_PL+S_^ML/MM^ and the ligand in solution Δ*G*_L+S_^ML/MM^ by NEQ switching from the MM PES to the ML/MM PES. Our workflow to calculate these terms is shown in Figure 3.

**Figure 3:**
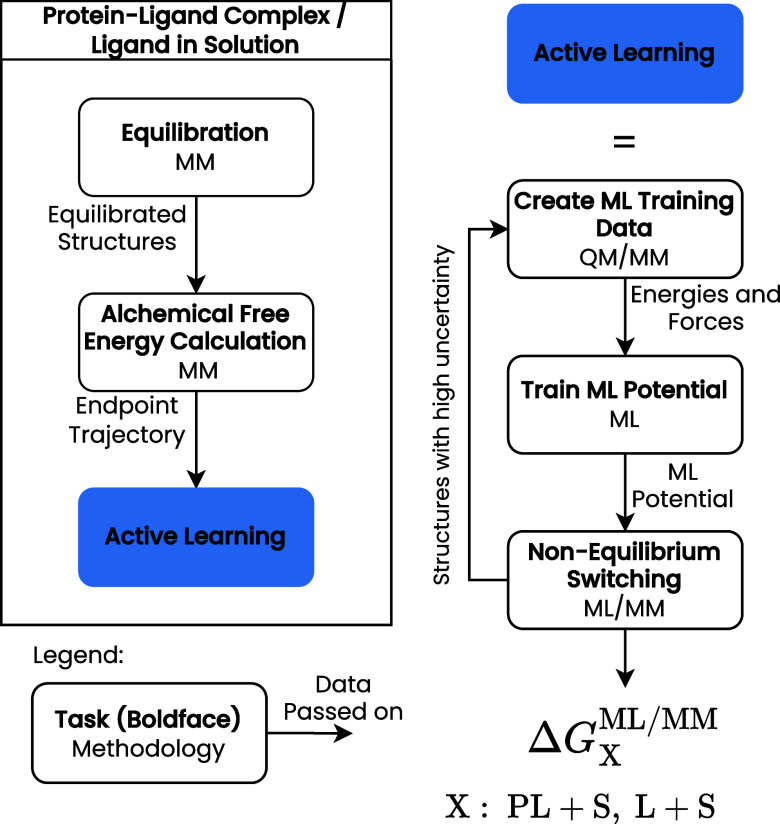
Workflow to determine the free energy of binding by ML/MM starting from a protein–ligand complex structure and a solvated ligand structure.

The first step is equilibrating the protein–ligand complex since we must perform AFE simulations, starting from the equilibrated system, to calculate the work required to transfer the ligand from the protein to vacuum and then solvate the ligand.

The initial training data set of the ML potential is based on structures from MD trajectories of both end states, the fully interacting ligand–protein complex, and the solvated ligand. The chosen structures need to be separated in time to reduce their correlation. QM/MM energies and forces are calculated as target properties for the initial training of the ML potential. Subsequently, NEQ switching simulations are carried out employing the ML potential to estimate the PES switching corrections Δ*G*_PL+S_^ML/MM^ and Δ*G*_L+S_^ML/MM^. Any structure encountered during these simulations that shows a high uncertainty in the energy or force predictions is collected. A selection of these structures, with a minimum distance in time to avoid too strong correlation, is recalculated by employing QM/MM and retrained by the ML potential. This procedure is iterated to systematically improve the quality of the ML potential.

### 3.3 Quantum Mechanics in Molecular Mechanics Embedding

In the hybrid QM/MM approach, the full system containing protein, ligand, and solvent (including solvated ions) is partitioned into the QM region *Q* and the remaining MM environment *E*. The interaction between both regions can be described by molecular mechanics or in a more elaborate approach using electrostatic embedding. We employ the latter approach, in which the electronic wave function of the QM region is polarized by the Coulomb potential of the MM point charges. The QM/MM energy is

4 $$E_{QM/MM}=E_{QM}+E_{elec}^{\mathrm{int}}+E_{MM}^{\mathrm{int}}+E_{MM}$$

where *E*_QM_ is the energy of the QM region, *E*_elec_^int^ is the electrostatic interaction between QM and MM region, *E*_MM_^int^ collects all nonelectrostatic interactions, and *E*_MM_ is the energy of the MM region.

The electrostatic interaction *E*_elec_^int^ is given as

5 $$E_{elec}^{\mathrm{int}}=\left\langle \Psi_{Q}\left|\sum_{A\in E}\frac{q_{A}}{\left|r-R_{A}\right|}\right|\Psi_{Q}\right\rangle +\sum_{I\in Q}\sum_{A\in E}\frac{Z_{I}q_{A}}{\left|R_{I}-R_{A}\right|}$$

Here, Ψ_*Q*_ denotes the wave function of the QM region. *q*_*A*_ is a point charge with the MM index *A* at position ***R***_*A*_ representing the electrostatic potential of the MM environment. *Z*_*I*_ is the nuclear charge of QM atom *I* at position ***R***_*I*_ in the QM region.

*E*_MM_^int^ includes all nonelectrostatic interactions between QM and MM regions, which are calculated by the MM force field. In this study, we restricted the QM region to the ligands because MM force fields are typically well optimized to describe protein residues and water. By contrast, parameters from general force fields may not be available or are considered less reliable in describing arbitrary ligands, making the description of the ligand a natural starting point to improve MM models. However, our choice in system partitioning also means that the interaction between protein and ligand is calculated with the MM force field, which may be the largest remaining uncertainty source in the model. To address this uncertainty, the QM region could be increased to contain protein atoms, reducing the reliance on classical MM force fields.

### 3.4 Machine Learning Potentials

As the ML potential architecture, we choose the second-generation HDNNP^31,32,56^ where the energy of a system with *N*_elem_ chemical elements and *N*_atom_^*m*^ atoms *n* of element *m* is constructed as a sum of atomic energy contributions *E*_atom,*n*_^*m*^. Feed-forward neural networks are employed to represent these atomic energy contributions, and, in this work, the neural network consists of two hidden layers with a linear output.^57^ The neural network input is vector **G**_*n*_^*m*^ of dimension *n*_G_, which describes the local atomic environment of atom *n*.

### 3.5 Element-Embracing Atom-Centered Symmetry Functions

Originally, the structural descriptor of HDNNPs is a vector of ACSFs.^80^ ACSFs are many-body representations of the interatomic distances and angles within a cutoff sphere. They fulfill the translational, rotational, and permutational invariances of the potential energy surface. Moreover, the ACSF vector size does not change with the atomic environment, making ACSF vectors applicable as input to pretrained feed-forward neural networks. Further, ACSFs enable the simulation of chemical reactions since they do not employ connectivities. However, each ACSF value represents only the distances or angles for a certain chemical element pair or triple. The scaling of the vector size is, therefore, unfavorable with respect to the number of chemical elements.

Element-embracing atom-centered symmetry functions (eeACSFs)^57^ solve this issue by including an explicit dependence on element information from the periodic table by the terms *H*_*i*,*j*_^rad^ and *H*_*i*,*jk*_^ang^ in the radial eeACSFs

6 $$G_{n,i}^{rad}=\sum_{j\neq n}^{N_{atom}}I_{i,j}^{rad}\times H_{i,j}^{rad}\times F_{i,j}^{rad}\left(R_{nj}\right)$$

and angular eeACSFs

7 $$G_{n,i}^{ang}=\sum_{j\neq n}^{N_{atom}}\sum_{k<j\wedge k\neq n}^{N_{atom}}I_{i,jk}^{ang}\times H_{i,jk}^{ang}\times F_{i,jk}^{ang}\left(\theta_{njk}\right)\times F_{i,j}^{rad}\left(R_{nj}\right)\times F_{i,k}^{rad}\left(R_{nk}\right)$$

respectively. The additional interaction type-dependent functions *I*_*i*,*j*_^rad^ and *I*_*i*,*jk*_^ang^ are introduced in the next subsection. In this work, the radial structure is described by

8 $$F_{i,j}^{rad}\left(R_{nj}\right)=\{\begin{array}{ll} \exp\left\{\eta_{i}\left[1-{\left(1-\frac{R_{nj}^{2}}{R_{c}^{2}}\right)}^{-1}\right]\right\} & \text{for},R_{nj}<R_{c}\\ 0 & otherwise \end{array}$$

which depends on the distances *R*_*nj*_ between the central atom *n* and the neighbor *j*. This function also dampens the eeACSF value and all of its derivatives smoothly to zero at the cutoff radius *R*_c_. We note that in the original ACSF and eeACSF approach, a separate cutoff function was applied on top of the radial function.^57,80^ For the angular structure representation, we apply the conventional function

9 $$F_{i,jk}^{ang}\left(\theta_{njk}\right)={\left[\frac{1}{2}+\frac{\lambda_{i}}{2}\cos\left(\theta_{njk}\right)\right]}^{\zeta_{i}}$$

with the angles θ_*njk*_ between atom *n* and the neighbors *j* and *k*. The application of various parameter values for η_*i*_ > 0, λ_*i*_ = ± 1, and ζ_*i*_ ≥ 1 in each eeACSF *i* can yield a structural fingerprint of atom *n*’s local atomic environment.

The initial approach for explicit element-dependence in ACSFs is given by weighted atom-centered symmetry functions.^81^ This descriptor employs the atomic numbers of the neighboring atoms to differentiate among various elements. To obtain a more balanced description of light and heavy elements and to exploit trends of the periodic table, the eeACSF vector includes different element properties *h* for the element-dependent term *H*

10 $$h_{i,j}\in \left\{1,n_{j},m_{j},d_{j},{\mathrm{n̅}}_{j},{\mathrm{m̅}}_{j},{\mathrm{d̅}}_{j}\right\}$$

These properties are the element’s period number in the periodic table *n*, the group number in the *s*- and p-block *m* (main group 1 to 8), and the group number in the d-block *d* (*d* = 0 for main group elements). For a balanced representation of all elements, these numbers are also counted in reverse order in the properties ${\mathrm{n̅}}_{j}:=X-n_{j}$ , ${\mathrm{m̅}}_{j}:=9-m_{j}$ , and ${\mathrm{d̅}}_{j}:=11-d_{j}$ ( d̅ = 0 for main group elements). In addition, element-independent eeACSF values are included in the eeACSF vector by setting *h*_*i*,*j*_ = 1. We note that extensions to the f-block can follow the scheme of the d-block. In this work, we apply the maximum period number *X* – 1 = 5; i.e., the heaviest element can be xenon. For d-block elements, we apply *m* = 2 and m̅ = 7.

In the radial eeACSFs, these element properties are divided by their maximal possible value *h*_*i*_^max^ of the element property *h* utilized in eeACSF *i*, to keep the element-dependent terms of each neighbor atom *j* between 0 and 1

11 $$H_{i,j}^{rad}=\frac{h_{i,j}}{h_{i}^{\max}}$$

The angular eeACSFs employ linear combinations of the element properties of neighbor *j* and *k* with hyperparameter γ_*i*_ = ± 1

12 $$H_{i,jk}^{ang}=\frac{\left|h_{i,j}+\gamma_{i}h_{i,k}\right|+\frac{1-\gamma_{i}}{2}C_{ijk}}{h_{i}^{\max}\left(\frac{1+\gamma_{i}}{2}+1\right)}$$

with

13 $$C_{ijk}=\{\begin{array}{ll} 0 & \text{for}{,h}_{i,j}=h_{i,k}=0\\ 1 & otherwise \end{array}$$

The value of the absolute linear combination is shifted by one if the difference (γ_*i*_ = −1) is applied. This approach ensures that each contribution is taken into account. If the elemental properties of both neighbors are equal to zero, this shift is not applied. The resulting value is divided by 2*h*_*i*_^max^ for sums (γ_*i*_ = 1) and by *h*_*i*_^max^ for differences (γ_*i*_ = −1). The GRP78–NKP1339 ligand contains five element types. A standard ACSF feature vector describing this system with five η_*i*_^rad^, two η_*i*_^ang^, two λ_*i*_, and three ζ_*i*_ would consist of 205 components, 180 of which are for the angular and 25 for the radial description. Conversely, the eeACSF vector would consist of 167 components, 35 of which are utilized for the radial part and 132 for the angular part. When applied to larger systems comprising more elements, the size advantage of eeACSF becomes even more pronounced.^57^

### 3.6 Representation of QM/MM Interactions in Element-Embracing Atom-Centered Symmetry Functions

ML potentials are constructed to obtain energies and atomic forces with first-principles quality but at an efficiency close to MM approaches. The most reasonable strategy to speed up QM/MM calculations with electrostatic embedding is, therefore, to learn the QM-related energy contribution *E*_QM_ + *E*_elec_^int^ and to evaluate *E*_MM_^int^ + *E*_MM_ by the MM approach (see eq 4). Since the ML potential descriptor takes into account only the local atomic environment, the long-range electrostatic interactions should be handled differently. Therefore, the electrostatic interactions between mixed QM–MM atom pairs are subtracted from the energy to be learned

14 $$E_{ML}=E_{QM}+E_{elec}^{\mathrm{int}}-\sum_{I\in Q}\sum_{A\in E}\frac{q_{I}q_{A}}{\left|R_{I}-R_{A}\right|}$$

In this work, the atomic charge of QM atom *I* is taken from the MM force field parameters. Therefore, a difference in the actual electrostatic interaction *E*_elec_^int^ exists, which needs to be covered by the ML potential. However, if the MM and QM charge distributions are similar, the difference should be small for large distances and the ML potential is able to account for the interaction within the cutoff radius. A more elaborate approach would be to learn environment-dependent atomic charges obtained from the QM calculations in a second ML model. Subsequently, these charges can be applied to calculate the long-range electrostatic interactions as in third-generation HDNNPs.^82^ We note that the MM atoms within the cutoff radius of a QM atom (environment region *E*′) contribute to the ML potential energy, and hence, their forces have MM and ML potential contributions.

In addition, we note that alternative approaches are trained on the difference between QM data and data from a semiempirical method.^33,34^ This so-called delta learning can increase the accuracy but has the drawback of requiring semiempirical method calculations for inference. To maximize efficiency, we avoid delta learning in our approach.

The energy to be learned still depends on the positions of the QM and MM atoms. However, the eeACSF vector is constructed to represent atomic environments in which all atoms are treated, employing the same approach. For ACSFs, this limitation was circumvented by introducing a new element type for MM atoms and multiplying the ACSF contributions by the respective atomic charges of the neighbors.^33^ To overcome this limitation also for eeACSFs, we propose an interaction-dependent term *I* in the eeACSFs, which is inspired by the spin-dependent ACSF approach.^83^ To differentiate the interactions from a QM atom to its QM neighbors *Q* and MM neighbors *E*, we introduce the atom type properties *t* ^*Q*^ and *t* ^*E*^. The former is one for QM neighbors and zero for MM neighbors, and the latter is vice versa. In this way, we can construct radial eeACSFs describing interactions only to QM atoms and only to MM atoms by the interaction-dependent term

15 $$I_{i,j}^{rad}\in \left\{t_{j}^{Q},t_{j}^{E}\right\}$$

The angular eeACSFs differentiate between interactions with two QM neighbors, one QM and one MM neighbor, and two MM neighbors

16 $$I_{i,jk}^{ang}\in \left\{t_{j}^{Q}\times t_{k}^{Q},t_{j}^{Q}\times t_{k}^{E}+t_{j}^{E}\times t_{k}^{Q},t_{j}^{E}\times t_{k}^{E}\right\}$$

Analogous to the element-dependent terms, different entries in the eeACSF vector can employ different interaction-dependent terms to represent all interactions.

The MM atoms enter energy *E*_QM_ + *E*_elec_^int^ by their electrostatic interaction with the QM atoms. Therefore, instead of the chemical element, the atomic charge is the only representative property of these atoms besides their positions. As a consequence, the element-dependent term in the radial eeACSF (eq 11) is replaced by

17 $$H_{i,j_{E}}^{rad}=\frac{q_{j_{E}}}{2q_{\max}}$$

if neighbor *j* is an MM atom. Its atomic charge *q*_jE_ is divided by two times the hyperparameter of the maximal possible absolute atomic charge *q*_max_. In this way, the maximal range of the *H* values is similar to those of interactions with QM neighbors, while larger absolute values of the atomic charges still lead to stronger interactions. For the angular eeACSFs, products of the terms from neighbors *j* and *k* are applied. This ansatz leads to

18 $$H_{i,jk_{E}}^{ang}=\frac{h_{i,j}q_{k_{E}}}{2h_{i}^{\max}q_{\max}}$$

if one neighbor is a QM atom and one is an MM atom (indices *j* and *k* can be interchanged), and to

19 $$H_{i,j_{E}k_{E}}^{ang}=\frac{q_{j_{E}}q_{k_{E}}}{2q_{\max}^{2}}$$

if both neighbors are MM atoms.

### 3.7 Uncertainty Quantification and Active Learning

The uncertainty of ML potentials has multiple sources, such as training data and model variance,^84^ and several approaches exist to reduce it.^84^ Uncertainty originating from insufficient training data can be alleviated by active learning strategies (explained below). An ensemble (or committee) of ML potentials that are trained on the same training data, but with differently initialized parameters, can reduce model variance and estimate the size of this uncertainty.^85–88^ Large model variance correlates with training data that are too sparse. The reason is that predictions are arbitrary for unknown structures due to the high flexibility of the ML model. In this way, missing training data can be identified, recalculated by the reference method, and trained by the ML potential. This process is called active learning.^59–62^ The retraining of the ML potential during active learning can be accelerated by continual learning strategies. This concept of so-called lifelong ML potentials^57^ exploits algorithms such as lifelong adaptive data selection and the continual resilient (CoRe) optimizer^57,89^ that enable efficient learning from new data and retaining of previous knowledge. In this way, the ML potential does not have to be retrained from scratch, and only a fraction of the previous training data must be employed to mitigate forgetting.

The energy of the ensemble model E̅ is given by the mean of the individual energies *E*_p_ of the *N*_MLP_ ML potentials *p*. The energy uncertainty ΔE̅ can be estimated by the sample standard deviation

20 $$\Delta \mathrm{E̅}=\max\{\overset{®}{RMSE\left(E^{test}\right)},{\left[\frac{c^{2}}{N_{MLP}-1}\sum_{p=1}^{N_{MLP}}{\left(E_{p}-\mathrm{E̅}\right)}^{2}\right]}^{1/2}\}$$

The scaling factor *c* can calibrate the uncertainty for a certain confidence interval. The minimum uncertainty is set to the mean of the root mean squared errors (RMSEs) of each ML potential’s test data. Analogous equations hold for the atomic force components.

## 4 Results and Discussion

Note that the technical computational details, including a description of the system preparation, the QM/MM modeling, the ML potential training, the alchemical free energy calculations, and the NEQ switching simulations, are given in the Supporting Information.

### 4.1 Relative Energy Distributions

The target quantity for training the ML potential is the energy of the QM region shifted by the electrostatic interaction between the MM and QM regions. To illustrate the QM energy values encountered during training, we plotted the distributions of *E*_QM_ in Figure 4. In these plots, we color-coded the energies calculated for structures from the end states of the classical AFE simulation (“MM Structures”) and the structures from active learning (“ML/MM Structures”).

**Figure 4:**
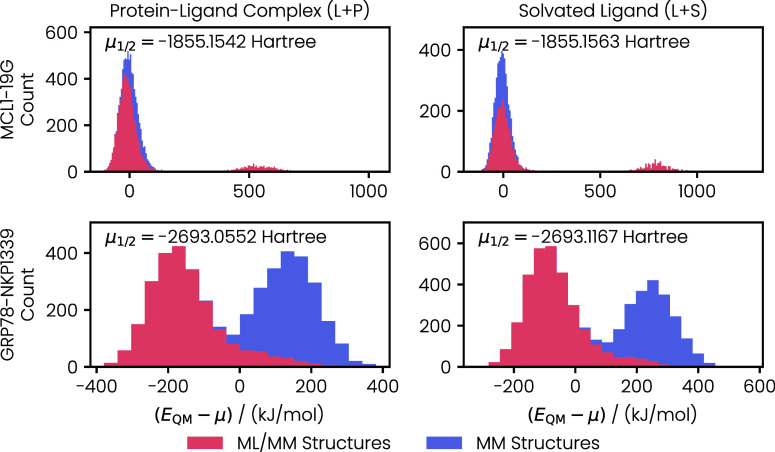
Energy distributions for the QM region calculated for the structures extracted from the end states of the classical AFE simulations and active learning. The distributions were shifted by their median μ_1/2_. The distributions for the structures from the MM trajectories are stacked on top of the distribution for the structures from active learning.

The *E*_QM_ distributions for both MCL1–19G end states (protein–ligand complex and solvated ligand) consist of a large Gaussian close to the median (0 kJ mol^–1^ in the plot) and a small peak at high energies. The high-energy structures were generated during the first iteration of active learning. They show short C–H and O–H bonds between atoms that are not covalently bonded according to the MM topology, as shown in the Supporting Information (Figure S1). For the solvated ligand, the energies of the Gaussian close to the median are distributed over the same interval, independent of whether they were calculated for structures extracted from the MM simulation or obtained during active learning. By contrast, we see a slight shift to higher energies for the MM structures of the protein–ligand complex. In general, we would expect that structures extracted from the active learning are likely to show lower energies than the structures from the MM trajectory because they were obtained during the ML/MM propagation, which allows for structural relaxation of the system according to the ML potential trained on the QM energies. Of course, this assumption does not apply to the first iterations of active learning, where the ML potential is highly uncertain and may produce high-energy structures.

Such high-energy structures are not encountered for the GRP78–NKP1339 protein–ligand complex or the NKP1339 solvated ligand. However, the shift between energy distributions from MM and active learning structures is very large for GRP78–NKP1339, as shown in Figure 4. For both end states, the distributions for the MM and active learning structures appear as Gaussian distributions, showing only a small overlap. The small overlap between the distributions suggests that the MM force field for GRP78–NKP1339 provides structures significantly different from those sampled during the ML/MM propagation. This difference is likely caused by the imperfect MM force field parameters that we generated for the Ru center in NKP1339. By contrast, the 19G ligand is purely organic and parameterized with the well-established GAFF force field in the MM simulations.

### 4.2 Accuracy of the Machine Learning Potential

For each system MCL1–19G, 19G, GRP78–NKP1339, and NKP1339, a separate HDNNP ensemble was trained. This approach of several smaller expert models instead of one larger general model leads to more flexibility in the learning process and potentially a higher accuracy-cost ratio in predictions.^90^ Each initial training was carried out on about 2 × 10^3^ training conformations from MM AFE simulations. Active learning, i.e., AFE simulations with current ML potentials, extraction, and recalculation of high-uncertainty structures, was applied to complete the sampling. Besides the reduction of high uncertainty predictions, the resulting binding free energy was monitored during active learning (Figures 6b and 7). With increasing reliability of the ML potentials, the binding free energy converged at a certain value. The final numbers of reference conformations *N*_conf_, i.e., both training and test conformations, are 9224 for MCL1–19G, 5967 for 19G, 4348 for GRP78–NKP1339, and 5234 for NKP1339, whereby each conformation includes *N*_*Q*_ = 44 QM atoms for MCL1–19G and 19G and *N*_*Q*_ = 35 QM atoms for GRP78–NKP1339 and NKP1339.

**Figure 5:**
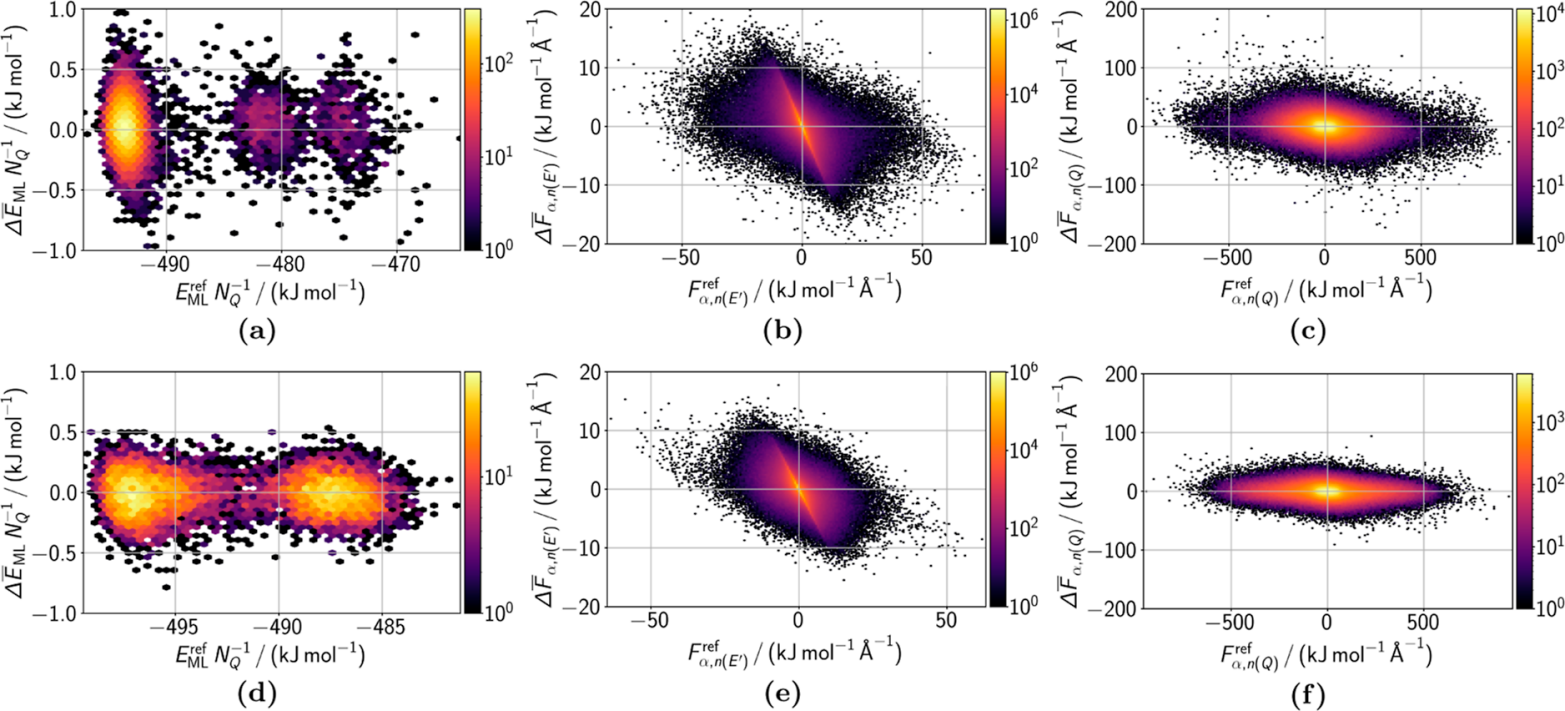
Deviations between the ensemble prediction and the QM-related reference data of (a–c) both MCL1–19G and 19G and (d–f) both GRP78–NKP1339 and NKP1339. The deviations are shown for (a,d) energies $\Delta {\mathrm{E̅}}_{ML}$ and atomic force components of (b,e) QM atoms $\Delta {\mathrm{F̅}}_{\alpha ,n\left(Q\right)}$ and (c,f) MM atoms represented by the ML potential $\Delta {\mathrm{F̅}}_{\alpha ,n\left(E^{\prime }\right)}$ as a function of the respective reference data *E*_ML_^ref^, *F*_α,*n*(*Q*)_^ref^, and $F_{\alpha ,n\left(E^{\prime }\right)}^{ref}$ . The color in this hexagonal binning plot visualizes the number of data points in a hexagon. Outside the shown error ranges are (a) 5 and (c) 6 data points.

**Figure 6:**
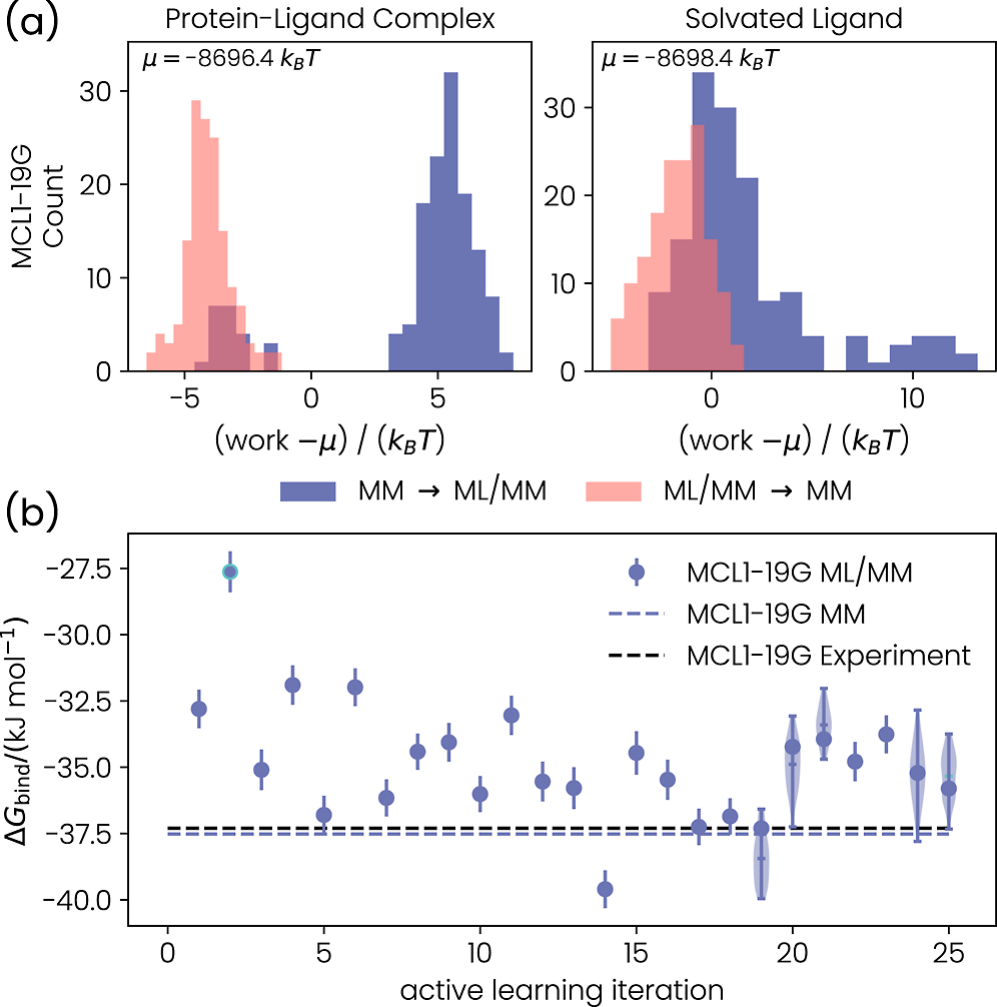
(a) Work distributions for the end states of MCL1–19G. The distributions were shifted by their mean μ for clarity. (b) Binding free energy Δ*G*_bind_ as a function of the active learning iteration for MCL1–19G. The error bars are the statistical error estimates from MBAR. Violin plots indicate explicit sampling of the uncertainty of the NEQ simulations. The mean and 1.5 times the interquartile range are shown in the violin plots.

If we compare the ranges of the reference data (Table 1) with the respective RMSEs of the prediction (Table 2), we observe that the errors are 2 orders of magnitude smaller than the values. This trend is true for all three properties: QM-related energies *E*_ML_; QM-related atomic force components of QM atoms *F*_α,*n*(*Q*)_; and MM atoms represented by ML potential *F*_α,*n*(*E*′)_.

**Table 1:** Ranges and Standard Deviations of *E*_ML_^ref^, *F*_α,*n*(*Q*)_^ref^, and $F_{\alpha ,n\left(E^{\prime }\right)}^{\mathrm{ref}}$

reference data	MCL1–19G	19G	GRP78–NKP1339	NKP1339
range(*E*_ML_^ref^/*N*_*Q*_)/kJ mol^–1^	26.916	31.771	16.478	17.868
std(*E*_ML_^ref^/*N*_*Q*_)/kJ mol^–1^	3.637	5.872	4.606	4.661
range(*F*_α,*n*(*Q*)_^ref^)/kJ mol^–1^ Å^–1^	1881.3	1787.0	1877.5	1760.7
std(*F*_α,*n*(*Q*)_^ref^)/kJ mol^–1^Å^–1^	86.8	95.0	134.9	129.5
range(*F*_α,*n*(*E′*)_^ref^)/kJ mol^–1^Å^–1^	146.12	136.95	125.46	110.91
std(*F*_α,*n*(*E′*)_^ref^)/kJ mol^–1^Å^–1^	1.84	2.72	1.88	2.61

**Table 2:** RMSEs of Individual HDNNPs and Ensembles for *E*_ML_, *F*_α,*n*(*Q*)_, and *F*_α,*n*(*E*′)_ ^a^

individual HDNNP RMSEs	MCL1–19G	19G	GRP78–NKP1339	NKP1339
*E*_ML_^train^*N*_*Q*_^–1^/kJ mol^–1^	0.190 ± 0.005	0.282 ± 0.012	0.181 ± 0.008	0.182 ± 0.007
*E*_ML_^test^*N*_*Q*_^–1^/kJ mol^–1^	0.186 ± 0.006	0.292 ± 0.012	0.179 ± 0.008	0.180 ± 0.010
*F*_α,*n*(*Q*)_^train^/kJ mol^–1^Å^–1^	12.3 ± 0.1	15.3 ± 0.2	10.3 ± 0.2	10.5 ± 0.2
*F*_α,*n*(*Q*)_^test^/kJ mol^–1^Å^–1^	11.9 ± 0.1	15.1 ± 0.1	10.0 ± 0.1	10.2 ± 0.2
$F_{\alpha ,n\left(E^{\prime }\right)}^{train}/kJ{\;mol}^{-1}{Å}^{-1}$	1.01 ± 0.02	1.78 ± 0.04	1.08 ± 0.02	1.56 ± 0.01
$F_{\alpha ,n\left(E^{\prime }\right)}^{test}/{kJ\;mol}^{-1}{Å}^{-1}$	1.00 ± 0.01	1.76 ± 0.04	1.07 ± 0.02	1.55 ± 0.01
ensemble RMSEs				
${\mathrm{E̅}}_{ML}N_{Q}^{-1}/kJ{\;mol}^{-1}$	0.158	0.237	0.152	0.157
${\mathrm{F̅}}_{\alpha ,n\left(Q\right)}/kJ\;{mol}^{-1}{Å}^{-1}$	10.57	13.20	9.01	9.214
${\mathrm{F̅}}_{\alpha ,n\left(E^{\prime }\right)}/kJ{\;mol}^{-1}{Å}^{-1}$	0.94	1.63	1.03	1.51
${\mathrm{F̅}}_{\alpha ,n\left(E^{\prime \prime }\right)}/kJ{\;mol}^{-1}{Å}^{-1}$	0.41	0.63	0.45	0.71

a The individual HDNNPs were evaluated on the final training data, i.e., without data declared to be redundant or inconsistent, and the test data. The respective values are determined as the mean over ten HDNNP results, and the given errors correspond to their standard deviation. The HDNNP ensembles were applied to all training and test data. ${\mathrm{F̅}}_{\alpha ,n\left(E^{\prime \prime }\right)}$ is the RMSE of QM-related atomic force components of MM atoms, which are not represented by the ML potential, but are at maximum 1 Å away from the represented atoms.

The training accuracy for all four systems (MCL1–19G, 19G, GRP78–NKP1339, and NKP1339) is in a similar range, proving the applicability of the same model parameterization for all systems. The differences in the RMSEs for the respective final training data and test data are very small for all systems (Table 2), revealing a good generalization of the ML potentials. Ensembling decreases the RMSE values for all *E*_ML_, *F*_α,*n*(*Q*)_, and *F*_α,*n*(*E*′)_ by a few percent. In general, the achieved accuracy is similar to that of previous approaches for ML potentials for QM/MM data.^33,34^ Therefore, the approach of subtracting the electrostatic interaction between point charges of QM and MM atoms from the data to be learned seems to be justified. More importantly, the accurate representation of eeACSFs extended for QM/MM data is demonstrated. Thus, with our approach, we can also construct efficient ML potentials for systems including many different chemical elements.

To examine whether a cutoff radius of *R*_c_ = 5 Å is sufficient for the representation of the QM-related atomic force components of MM atoms, we calculated the RMSE of MM atoms which are between 5 and 6 Å away from the QM atoms. These atoms are not represented by the ML potential, and hence, the QM-related force prediction will be zero. We excluded atoms further away because their interaction with the QM region is smaller, thus reducing the RMSE. However, Table 2 shows that these RMSEs of ${\mathrm{F̅}}_{\alpha ,n\left(E^{\prime \prime }\right)}$ are less than half the size of the values for ${\mathrm{F̅}}_{\alpha ,n\left(E^{\prime }\right)}$ . The latter are also 1 order of magnitude smaller than the RMSEs for the forces of QM atoms ${\mathrm{F̅}}_{\alpha ,n\left(Q\right)}$ . Therefore, the applied cutoff radius is sufficient for the representation of the QM-related forces of the MM atoms. This small cutoff radius is also a plus of the ansatz to subtract the electrostatic interaction between point charges of QM and MM atoms from the data to be learned.

Figure 5a shows the QM-related energy prediction errors of HDNNP ensembles as a function of the reference energies for the combined MCL1–19G and 19G data. Most of the reference energies are concentrated in an interval of about 4 kJ mol^–1^ per atom, which appears to be reasonable due to fluctuations in the kinetic energy at 300 K as well as the different chemical environments of the QM ligand atoms. The prediction errors of these data points are symmetrically centered around zero, as expected. In addition, there are some high-energy structures, which originate from active learning (see Figure 4). However, for GRP78–NKP1339 and NKP1339, the reference energies are concentrated in two intervals. While the interval at higher energies includes mainly structures from the initial MM sampling, the interval at lower energies originates from active learning. Hence, the sampled conformation spaces by MM and ML/MM differ. Moreover, from this observation, we expect that the binding free energy difference between the MM and ML/MM representation will be larger for GRP78–NKP1339 than for MCL1–19G. This is in line with the fact that MCL1–19G is a well-studied benchmark system for MM, while GRP78–NKP1339 contains a transition metal atom, which is often problematic for MM.

The QM-related atomic force component error distributions for both systems look similar (Figure 5b,c,e,f). While the force errors of the QM atoms are well centered around zero, the force errors of some part of the MM atoms show a trend for small force values. As a result of this trend, the affected forces are predicted as zero, while their reference values are slightly above or below zero. This behavior is primarily caused by MM atoms located at the edge of the cutoff sphere of certain QM atom(s), which are described very little by the eeACSF vector. Therefore, calculating their derivatives is difficult, and the best compromise is to predict the mean value, i.e., zero. However, these force errors are below the RMSE of the force prediction error and contribute minimally to the overall error, indicating a sufficiently large cutoff radius. For larger values of *F*_α,*n*(*Q*)_^ref^, the prediction error distribution again centers around zero.

### 4.3 Free Energies of Binding

#### 4.3.1 MCL1–19G

Our prediction of the absolute binding free energy Δ*G*_bind_^MM^ based on the classical AFE simulations is −37.5 kJ mol^–1^ ± 0.4 kJ mol^–1^, matching the experimental estimate (−37.3 kJ mol^–1^ ± 0.1 kJ mol^–1^)^64^ nearly perfectly. Note that such close agreement with the experimental data is not always the case. Previous studies using absolute AFE calculations on sets of multiple ligands have reported an RMSE to experiment of around 6–12 kJ mol^–1^ depending on the system.^91–94^

The work distributions from the NEQ switching simulations from the MM to the ML/MM PESs for MCL1–19G are shown in Figure 6a. Overall, the work distributions for MCL1–19G are narrow, showing standard deviations of only 3.0 *k*_B_ *T* and 1.0 *k*_B_ *T* for the backward and forward switches of the protein–ligand complex, respectively. We calculated similarly low standard deviations for the work distributions of the solvated 19G ligand (forward switch: σ = 3.7 *k*_B_ *T*, backward switch: σ = 2.0 *k*_*B*_ *T*). Furthermore, the forward and backward switches show significant overlap in both cases. The low standard deviations and the overlap indicate that the relatively short switching time of 10 ps was sufficient.

The work distributions of the forward switches of the solvated 19G ligand and the MCL1–19G protein–ligand complex show two distinct peaks, which disappear in the backward switches. By investigating the trajectories from the switching simulations, we found that the individual peaks correspond to different hydrogen­(H)-bonding partners of the 19G ligand’s carboxyl group. The peaks at low work (work – μ < 0) are associated with H-bonds to solvent molecules, while the peak at high work (work – μ > 0) is associated with an H-bond to the sulfur atom in 19G. The H-bond with sulfur is destabilized in the ML/MM compared to the MM simulation, and we observe only H-bonds to solvent molecules after switching. Therefore, it requires more work to switch from MM to ML/MM if starting from a 19G conformation H-bonded to sulfur. In addition, all backward-switching simulations start from conformations with H-bonds to the solvent, which do not switch to sulfur during the simulation time, leading to a single peak for the backward switches.

To illustrate the effect of the active learning procedure on our ML/MM binding free energy estimate, we plotted Δ*G*_bind_^ML/MM^ as a function of the active learning iteration in Figure 6b for MCL1–19G. Furthermore, we also show the experimental estimate and the estimate from the initial MM AFE calculations as dashed lines. We did not obtain a free energy estimate for the zeroth iteration ML potential, i.e., the ML potential purely trained on the MM structures. The simulation stopped because too many structures had already been collected for active learning. Our binding free energy estimate shows a relatively widespread range of values for iterations one to six. For the higher iterations, the binding free energy estimate is scattered close to 35 kJ mol^–1^. Since the variance between iterations was significantly larger than the error estimate provided by MBAR, we sampled the binding free energy by running five additional NEQ switching simulations for each end state using the ML potential of active learning iterations 20 and 19. This sampling procedure provided 36 estimates of the binding free energy for each active learning iteration. The distributions of these estimates are illustrated as violin plots. The distributions are significantly broader than the MBAR error estimates. Furthermore, they show only a little overlap, indicating that the active learning had not converged. Afterward, we increased the number of epochs for the MLP optimization from 1000 to 5000 and continued active learning up to iteration 25. Because of the high overlap between the Δ*G*_bind_^ML/MM^ distributions for iterations 24 and 25, we considered the active learning procedure converged.

Because of the large spread of the free energy estimates, we calculated our final value of Δ*G*_bind_^ML/MM^ as the mean of the distribution with twice its standard deviation as an error estimate of ⟨Δ*G*_bind_^ML/MM^⟩ = -35.3 kJ mol^–1^ ± 1.8 kJ mol^–1^. This final result is close to the experimental estimate of −37.3 kJ mol^–1^ ± 0.1 kJ mol^–1^ if we include the error ranges. Furthermore, we note that the protonation state of the ligand is unclear. The carboxylic acid group may be deprotonated in solution, which we did not simulate.

Furthermore, the accuracy of our prediction workflow can be systematically improved further by multiple strategies: (i) Currently, we restricted the QM region to the ligand. However, our approach can easily extend the QM region to the protein, leading to a more accurate description of the protein–ligand interaction. (ii) The QM region can be described with a more accurate electronic structure method, such as a hybrid exchange–correlation functional or by exploiting multilevel QM/QM embedding, as demonstrated in refs 95 and 96 .

To characterize the difference between the structural ensembles produced by the MM and ML/MM potentials for the MCL1–19G system, we compared the ligand dihedral angle distributions produced by simulations with the two potentials, both in the bound and unbound states (Figure 8a). As we did not have a converged equilibrium simulation with the ML/MM potential, we instead used an ensemble consisting of the final structure of each ML/MM equilibration simulation from the middle of the NEQ switching cycle as an approximation of the equilibrium ensemble. We found that the MM and ML/MM potentials produce similar dihedral angle distributions for the 19G ligand in the bound state (complex), while there were slight shifts in the populations in the unbound state (solvent). This is consistent with the narrower work distributions from NEQ switching simulations observed for the protein–ligand complex and is likely related to the higher level of ligand structural heterogeneity in the unbound state, which is also reflected in the dihedral angle distributions. Differences in the torsion potentials between the MM and ML potentials could explain why the models produce different values of Δ*G*_bind_.

**Figure 7:**
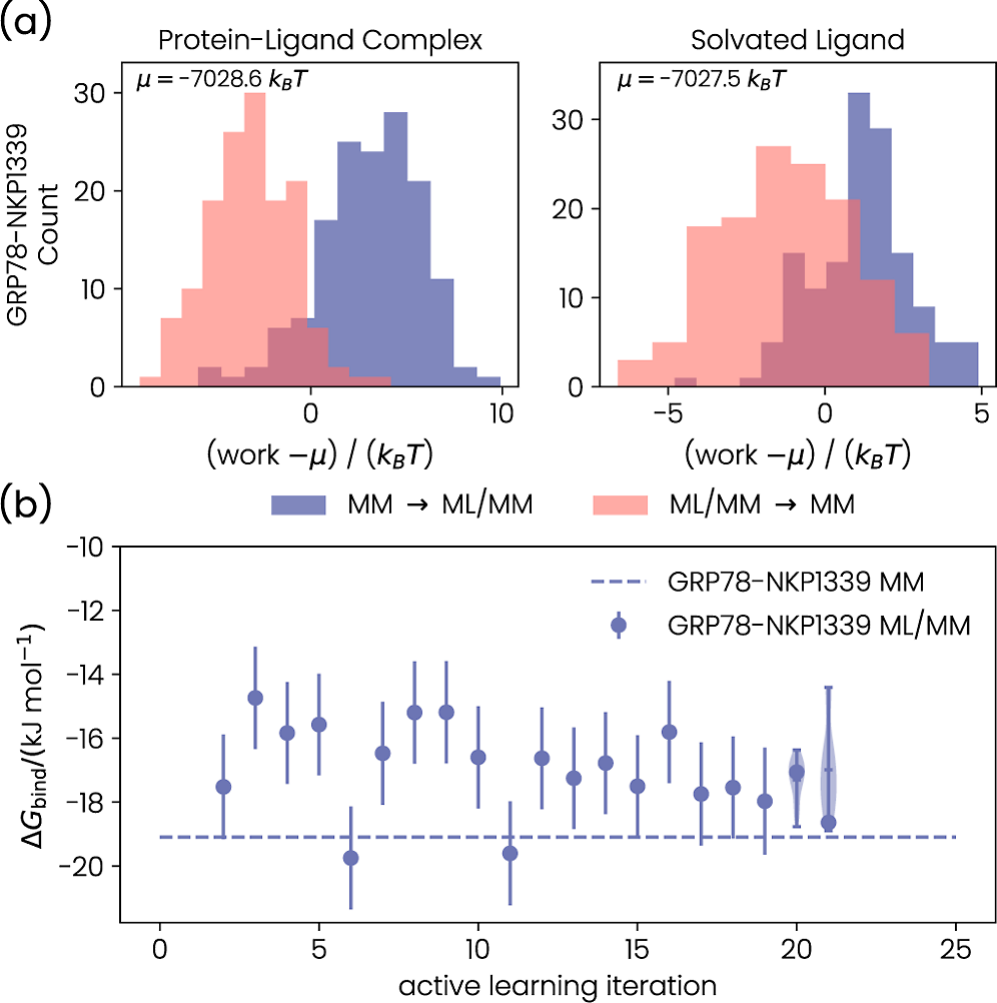
(a) Work distributions for the end states of GRP78–NKP1339. The distributions were shifted by their mean μ for clarity. (b) Binding free energy *G*_bind_ as a function of the active learning iteration for GRP78–NKP1339. The error bars are the statistical error estimates from MBAR. Violin plots indicate explicit sampling of the uncertainty of the NEQ simulations. The mean and 1.5 times the interquartile range are shown in the violin plots.

**Figure 8:**
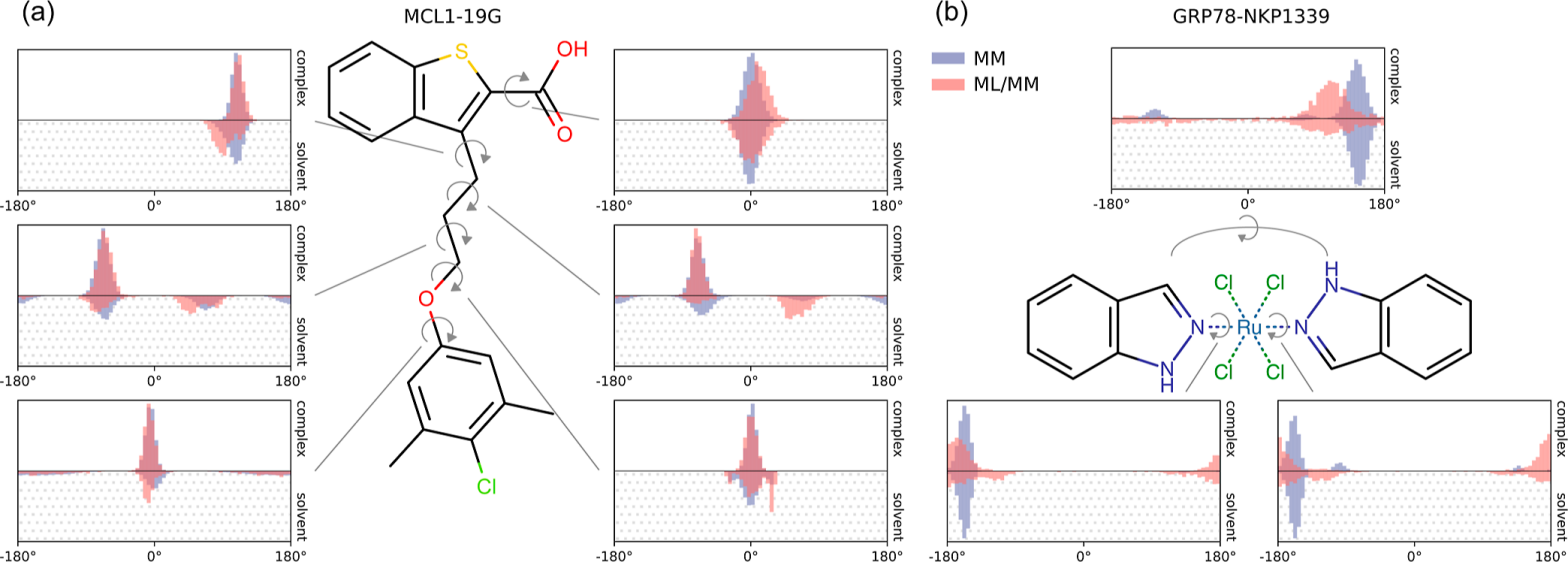
Dihedral angle distributions for (a) 19G in the MCL1–19G complex and in solution and (b) NKP1339 in the GRP78–NKP1339 complex and in solution. Distributions were calculated from simulations with the MM potential (blue) and ML/MM potential (red). ML/MM distributions were calculated over parallel NEQ switching simulations, using the final structure of the ML/MM equilibration step.

For comparison, we also performed end-state corrections using NEQ switching simulations from MM to an ANI-2x/MM potential, where the 19G ligand is described by ANI-2x. ANI-2x is a transferable MLP trained to reproduce QM calculations.^77^ Six replicate NEQ switching calculations to the ANI-2x/MM potential resulted in ⟨Δ*G*_bind_^ANI‑2x/MM^⟩ = -33.14 kJ mol^–1^ ± 0.7 kJ mol^–1^. Consistent with the results using our MLP, the correction to Δ*G*_bind_ is positive, although ANI-2x results in a slightly larger correction. To investigate the effect of the ANI-2x correction on the structural ensemble of the ligand, we also calculated the ligand dihedral angle distributions from these simulations (Figure S2). We found that, compared with the dihedral angle distributions produced by our MLP, the ANI-2x distributions were generally more similar to the MM distributions. As ANI-2x gave rise to a larger correction of Δ*G*_bind_ than our MLP, one might expect a correspondingly larger correction of the torsion potentials of the ligand with ANI-2x, but this does not seem to be the case.

#### 4.3.2 GRP78–NKP1339

The results above serve as a test case where we expect that a well-parameterized classical force field may perform well. We now proceeded to study a more complex case involving a ligand with a transition metal. For the GRP78–NKP1339 protein–ligand complex, the classical MM AFE simulation predicts a binding free energy of Δ*G*_bind_^MM^ = −19.1 kJ mol^–1^ ± 1.5 kJ mol^–1^. Note that no experimental estimate is available for this protein–drug complex.

The work distributions calculated with the final ML potential for GRP78–NKP1339 are shown in Figure 7a. There is significant overlap between the forward and backward work distributions for both end states (protein–ligand complex and solvated ligand). Furthermore, the distributions have only a single peak, suggesting that there is no qualitative difference between ligand conformers, which was encountered for MCL1–19G.

In Figure 7b, we show convergence of the binding free energy as a function of the active learning iteration. We did not obtain free energy estimates with the initial (purely based on MM structures) ML potential and the first ML potential from active learning. These runs did not provide binding free energy estimates because the calculations were stopped after too many structures were assigned to active learning, as discussed in Section S1. The active learning for the solvated ligand naturally converged after the 14th iteration since no additional structures were considered for training. For the protein–ligand complex, we continued the active learning until the 20th iteration, after which the number of new structures dropped below 10. We then retrained the MLP with 5000 instead of 1000 epochs and performed the final NEQ switching simulations.

To estimate the uncertainty caused by the NEQ procedure of our binding free energy estimate, we sampled the end-state corrections with five additional NEQ simulations for each end state. The resulting Δ*G*_bind_^ML/MM^ distribution is shown as violin plots for the active learning iterations 20 and 21 in Figure 7b. The distribution is narrower compared to the distribution obtained for MCL1–19G. We calculated our final Δ*G*_bind_^ML/MM^ estimate as ⟨Δ*G*_bind_^ML/MM^⟩ = −17.0 kJ mol^–1^ ± 2.6 kJ mol^–1^, where we used twice the standard deviation of the underlying distribution as an error. This estimate for Δ*G*_bind_ differs from the MM estimate (Δ*G*_bind_^MM^ = −19.1 kJ mol^–1^) by 2.1 kJ mol^–1^. However, the error bars for both results still overlap. The fact that the MM and ML/MM estimates are close is somewhat surprising because the QM energies were significantly different for the initial MM structures and the active learning structures (see Figure 4). The energy gap between MM and active learning structures suggests there are qualitative differences between them. However, these differences do not appear to affect the ability of NKP1339 to bind to GRP78 since the binding pocket provides sufficient flexibility to accommodate both equilibrium conformations (ML/MM and MM). The large error introduced in the MM description largely cancels during the calculation of the binding free energy since it affects both the solvated ligand and the protein–ligand complex in a similar way.

We also characterized the difference between the structural ensembles produced by the MM and ML/MM potentials for the GRP78–NKP1339 system. We found that the coordination geometry around the Ru remains unchanged (Figure S3); the ML/MM potential results in slightly increased Cl–Ru distances and decreased indazole–Ru distances (Figure 9). Additionally, the ML/MM potential shifts the angle of the indazoles slightly, so they are aligned with the chlorides and closer to 90° staggered with respect to each other (Figure 8b). In the unbound state, the ML/MM potential allows for more freedom in rotation of the Ru–indazole bond. This is consistent with the distance distributions between the chlorides and nearest hydrogens on the indazoles, which are generally broader with the ML/MM potential, but also show an increased population of shorter distances corresponding to the alignment of the indazoles with the chlorides (Figure S4). As for MCL1–19G, these results suggest that there are differences between the MM and ML potentials, which could affect the Δ*G*_bind_.

**Figure 9:**
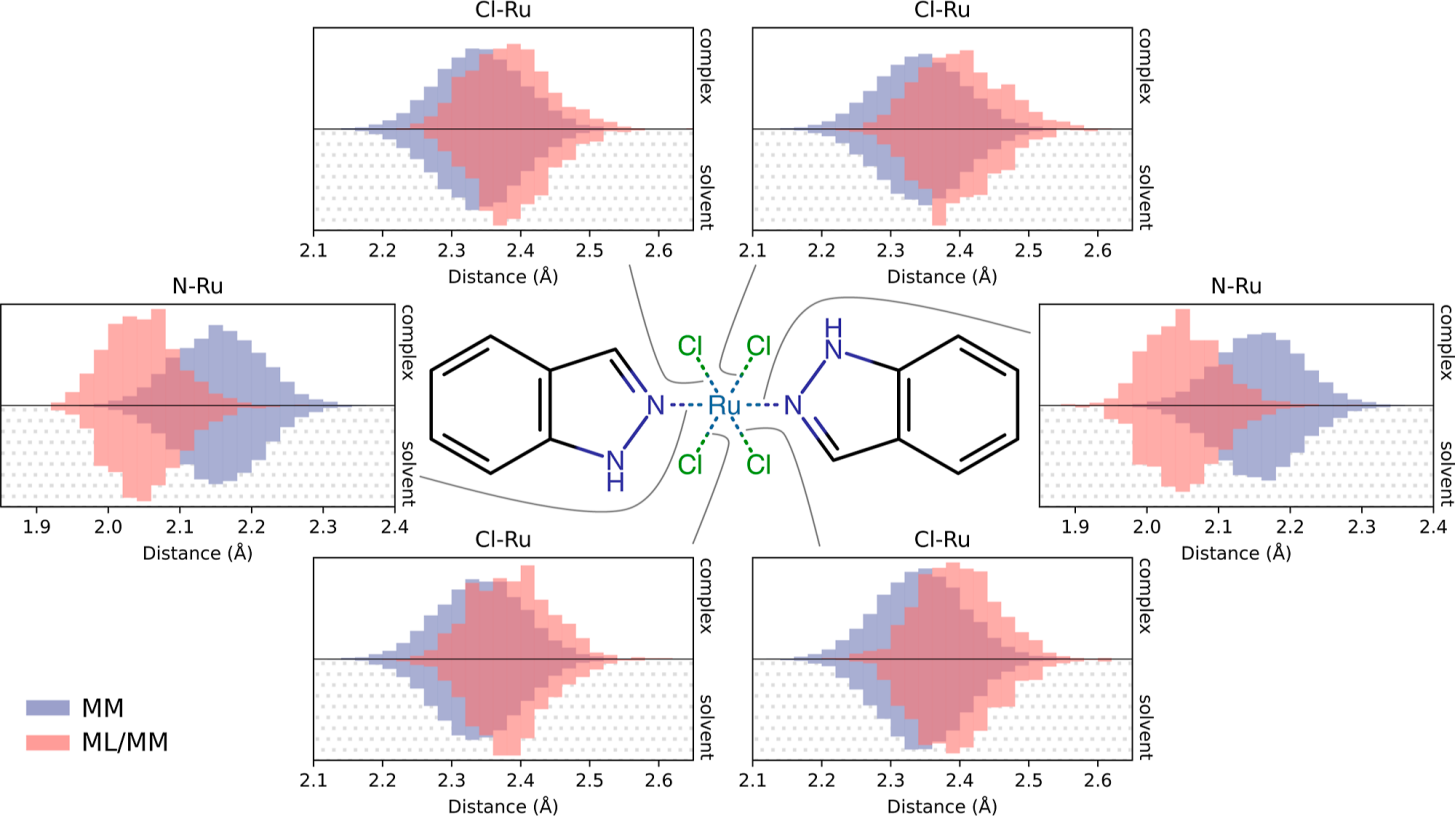
Distance distributions around the Ru center for NKP1339 in the GRP78–NKP1339 complex and in solution. Distributions were calculated from simulations with the MM potential (blue) and ML/MM potential (red). ML/MM distributions were calculated over parallel NEQ switching simulations, using the final structure of the ML/MM equilibration step.

It is interesting to investigate the computational resources required by our pipeline. On contemporary commercial-off-the-shelf hardware (i.e., a computing node with two AMD EPYC 9954 CPUs with a total of 128 cores with 3 GHz base frequency and 512 GB of RAM), one QM/MM calculation on the MCL1–19G complex took about 255 s on a single core and 48 s on 16 cores. Hence, with 1000 CPU cores available, the calculation of 5000 reference data points can be done in about 1 h. Training the machine learning potential (with 1000 epochs) takes about 751 s. This value rises to 1151 and 1485 s when increasing the number of training structures to 3000 and 4000, respectively. Finally, in the NEQ switching, we can simulate about 0.9 ns per day of computing time on six CPU cores, such that a switch of 10 ps takes about 16 min. Therefore, all 150 forward and backward switches of an NEQ run and the 10 ps equilibration between switches can be run within 48 min of computing time using 900 CPU cores.

## 5 Conclusions

We presented a complete workflow to predict the absolute binding free energies of protein–ligand complexes. Our workflow employs state-of-the-art AFE simulations to predict the binding free energy by using MM force fields. The MM free energies are then corrected through NEQ switching simulations, describing the work required to switch from the MM PES to a QM/MM PES. To efficiently perform the NEQ switching simulations, we used ML potentials trained explicitly for protein–ligand systems. We exploited active learning to minimize the number of QM/MM reference calculations required for our workflow and ensured that the ML potentials provided an accurate description of the QM/MM energies and forces. Furthermore, we implemented our workflow as a distributed computing framework to use computational resources in multiple high-performance computing centers and reduce the time required to run reference calculations for the ML potentials.

During the development of the workflow, we ensured that it could be systematically improved by using more accurate electronic structure methods or multilevel QM/QM embedding, as shown in refs 95 and 96 . As a second dimension to increase the workflow’s accuracy, the QM region in the QM/MM embedding could be extended to the protein, increasing the accuracy of the description of the protein–ligand interaction, by reducing its reliance on MM point charges and Lennard-Jones parameters. Furthermore, our workflow can be applied to any ligand–protein system, independent of the elementary composition. This is possible because the element embracing symmetry functions used as a descriptor in the ML potential can treat many different chemical elements efficiently, avoiding the dimensions of the descriptor growing out of proportion if many chemical elements are present. Additionally, we do not rely on pretrained ML force fields, which are restricted to a small selection of elements. Therefore, ligands containing transition metal atoms can also be described easily. Long-range electrostatic interactions could be described by the ML potential by training it on the difference of the electrostatic interactions in the QM/MM and MM models. Since the electrostatic interaction can be well described by the point-charge interaction in MM for large distances, this difference is significant only for the local environment of the QM region. Therefore, we could show that the ML potential can accurately reproduce this difference by relying on a descriptor that relies only on the local environment of each atom. We showed that our final ML approach is highly accurate, requiring only the position and elements of the system as input and that it is highly efficient such that it can be applied in NEQ switching simulations.

We demonstrated our workflow for two protein–ligand complexes. The MCL1–19G complex served as a benchmark to establish the workflow’s reliability because experimental data was available. For this system, the end-state corrections were found to be close to the already reliable results from MM. For the second test system, the complex of GRP78 and the anticancer drug NKP1339, we found that our initial MM parameterization provided structures with significantly higher QM energy than those obtained during active learning. The drug NKP1339 contains a Ru atom for which no established MM force field parameters were available. Therefore, the initial MM force field provided an inadequate description, which was corrected by our approach. This demonstrates that our workflow is not restricted to a specific set of elements and can be applied to a wide range of systems. Since the errors in the MM force field were entered into the description of the solvated ligand and the protein–ligand complex equally, the final binding free energies predicted by our ML/MM and the initial MM approach were close.

## Supplementary Material



## Data Availability

The machine learning potentials, the databases containing all QM/MM energies, and the software are available on ERDA.^97^

## Notes

Computational details, supporting results, and archive on the platform ERDA containing all software and data necessary to reproduce the results of this study^97^ (PDF)
