# Supplementary material for: Machine Learning-Enhanced Calculation of Quantum-Classical Binding Free Energies
Source: J Chem Theory Comput. 2025 Aug 5;21(16):8182–98. doi: 10.1021/acs.jctc.5c00388 (PMC12392446; doi:10.1021/acs.jctc.5c00388)
Supplement: Supplementary file 1 [file ct5c00388_si_001.pdf]

Supporting Information: Machine Learning Enhanced Calculation of Quantum-Classical Binding Free Energies

Moritz Bensberg,[1,*] Marco Eckhoff,[1,*] F. Emil Thomasen,[2,*] William Bro-Jørgensen,[3,*] Matthew S. Teynor,[3,4] Valentina Sora,[5] Thomas Weymuth,[1] Raphael T. Husistein,[1] Frederik E. Knudsen,[2] Anders Krogh,[6,†] Kresten Lindorff-Larsen,[2,‡] Markus Reiher,[1,§] and Gemma C. Solomon[3,4,¶]

[1] *ETH Zurich, Department of Chemistry and Applied Biosciences, Vladimir-Prelog-Weg 2, 8093 Zurich, Switzerland.*
[2] *University of Copenhagen, Department of Biology, Linderstrøm-Lang Centre for Protein Science, Ole Maaløes Vej 5, DK-2200, Copenhagen N, Denmark.*
[3] *University of Copenhagen, Department of Chemistry and Nano-Science Center, Universitetsparken 5, DK-2100, Copenhagen Ø, Denmark.*
[4] *University of Copenhagen, Niels Bohr Institute, NNF Quantum Computing Programme, Blegdamsvej 21, DK-2100 Copenhagen Ø, Denmark.*
[5] *University of Copenhagen, Department of Computer Science, Universitetsparken 1, DK-2100, Copenhagen Ø, Denmark.*
[6] *University of Copenhagen, Department of Computer Science, Universitetsparken 1, DK-2100, Copenhagen Ø, Denmark and Center for Health Data Science, Department of Public Health.*

(Dated: March 5, 2025)

S1. SUPPORTING COMPUTATIONAL DETAILS

S1.1. System Preparation

We prepared the MCL1-19G and GRP78-NKP1339 systems using the Python packages pdbcraft (`https://github.com/Center-for-Health-Data-Science/pdbcraft`) and openmmwrap (`https://github.com/Quantum-for-Life/openmmwrap`) to streamline the preparation steps.

The Amber99sb*ILDN force field [1] was applied to parametrize the MCL1 and GRP78 proteins. We parametrized the 19G ligand employing the GAFF 2.11 force field [2, 3] and the OpenFF toolkit [4]. Water was represented by the TIP3P force field [5].

We generated initial force field parameters for the NKP1339 ligand by creating a system-focused atomic model (SFAM) without hydrogen bonding. The SFAM force field parameters were obtained in a fully automated fashion with the parametrization procedure implemented in SCINE Swoose[6–9]. For the required DFT reference calculations, PBE0-D3BJ/def2-TZVP[10–13] was applied. The initial FF parameters were obtained for the fully optimized structure of NKP1339, *i.e.*, no bond distances or angles were kept fixed. We modified the resulting force field parameters to account for the square planar geometry of the chlorine atoms and the interaction between the indazole groups and the chlorine atoms. The square planar geometry was parameterized by removing the harmonic angle term for Cl-Ru-Cl angles from the SCINE Swoose output and adding a custom angle term of the form

$$k_{\mathrm{Cl-Ru-Cl}}\left(1+\cos(4\theta-180^\circ)\right)+0.04\epsilon_{\mathrm{LJ}}\left(\left(\frac{\sigma_{\mathrm{LJ}}}{2d_{\mathrm{LJ}}\sin(0.5\theta)}\right)^{12}-\left(\frac{\sigma_{\mathrm{LJ}}}{2d_{\mathrm{LJ}}\sin(0.5\theta)}\right)^{6}\right) \quad (1)$$

with $k_{\mathrm{Cl-Ru-Cl}} = 42.356$ kJ mol^{-1}, θ as the Cl-Ru-Cl angle, and the Lennard-Jones parameters for Cl-Ru of $\epsilon_{\mathrm{LJ}} = 1.375$ kJ mol^{-1}, $\sigma_{\mathrm{LJ}} = 0.378$ nm, and $d_{\mathrm{LJ}} = 0.236$ nm. This gives a periodic force with potential energy minima at $\theta = 90^\circ$, 180°, and 270°, and importantly not at $\theta = 0^\circ$. The interaction between the indazole groups and the chlorine atoms was parameterized by adding a proper periodic torsion force for Cl-Ru-N-C dihedral angles with a periodicity of 4, a phase of 234.5°, and a force constant of 4.999 kJ mol^{-1}. This does not capture the interaction between the indazole groups, which would require calculating interactions between atoms that are separated by four or more bonds.

* Authors contributed equally.
† akrogh@di.ku.dk
‡ lindorff@bio.ku.dk
§ mreiher@ethz.ch
¶ gsolomon@chem.ku.dk

For the MCL1-19G system, we downloaded the 4HW3 [14] structure from the Protein Data Bank [15]. We removed crystallographic water and added missing heavy atoms to the protein and missing hydrogen atoms to the protein and the ligand. We capped the protein at both termini.

For the GRP78-NKP1339 system, we downloaded the 3LDO [16] structure from the Protein Data Bank and kept only the protein, removing the ANP compound and crystallographic water. We added missing hydrogen atoms to the protein and capped it at both termini. Finally, we merged the protein and the handcrafted structure of the NKP1339 ligand.

Both protein-ligand systems were solvated in a box of water. Each system was first neutralized with Na^+ or Cl^- ions, and then pairs of these ions were added to reach a salt concentration of 150 mM. Subsequently, we minimized the energy of each system before performing two rounds of equilibration. The first equilibration was a 2 ns run in the canonical (NVT) ensemble employing the Langevin integrator implemented in OpenMM [17, 18] and harmonic position restraints for the ligand. The second equilibration was carried out for 2 ns in the isothermal–isobaric (NpT) ensemble without restraints. The Monte Carlo (MC) barostat was applied for pressure coupling [18–20]. All equilibrations were performed at 298 K with time steps of 1 fs.

To set up an alchemical free energy (AFE) simulation of a ligand in solution, we solvated the ligand in a box with box vectors taken from the respective equilibrated protein–ligand complex. The system was again neutralized with Na^+ or Cl^- ions and the salt concentration was adjusted to a concentration of 150 mM.

Before running AFE simulations with Yank, all systems were automatically energy minimized and equilibrated using the default settings of Yank [21]: 1000 steps of energy minimization with the FIRE minimizer [22] followed by one iteration of the production simulation protocol described in Section 4.4.

S1.2. Quantum Mechanics in Molecular Mechanics Embedding

The quantum mechanics (QM) region in the QM/MM calculations for the training data generation was described employing Kohn–Sham density functional theory (DFT), Perdew, Burke, and Ernzerhof's exchange–correlation functional known as PBE [23], and the def2-SVP basis set [24]. The long-range dispersion interaction was included with Grimme's D3 dispersion correction [11] using Becke–Johnson damping [12]. The embedded DFT calculation was polarized by the MM point charges, as described in Section 3.3. We note that only the point charges in the simulation box were included in this electrostatic embedding, and the interaction with the periodic box images was assumed to be negligible. To reduce the error of this approximation, the molecules in the simulation box were translated by the periodic box vectors such that the first atom of the QM region was at the box's center. The QM/MM calculations were performed with the program SCINE Swoose[6, 9, 25] using an interface to the quantum chemistry program Turbomole[26, 27].

The MM region and the non-electrostatic interaction between the QM and MM regions were described employing the same Amber-type force field as in the system preparation. The MM contributions to the energies and forces were calculated using the program SCINE Swoose.

A central database facilitated the large number of QM/MM single-point calculations required for training the ML potentials[9, 28]. This database contained all molecular structures from the initial MM simulations and active learning, as well as the input and output of the QM/MM calculations.

S1.3. Machine Learning Potentials

We applied an ensemble size of $N_{\mathrm{MLP}} = 10$ HDNNPs, with the uncertainty scaling factor $c = 2$. The feed-forward neural networks of every element (H, C, N, O, S, Cl, and Ru) in all HDNNPs employed an input layer with $n_G = 174$ neurons, three hidden layers with $n_1 = 120$, $n_2 = 72$, and $n_3 = 48$ neurons, and an output layer with one neuron. A scaled hyperbolic tangent [29] was applied as the activation function. The weights were initialized according to the scheme in Reference [29]. The cutoff radius of the eeACSFs was set to $R_{\mathrm{c}} = 5\,\text{Å}$. All other eeACSF parameter values are provided in Tables S1 and S2.

The energy contributions of neutral free atoms in their lowest spin state were removed from the QM/MM reference data for the QM atoms (see Table S3 in the Supporting Information). Structures including atomic forces higher than $10\,\mathrm{eV\,Å^{-1}}$ were excluded before training. For each HDNNP, the reference data was split randomly into 90% training structures and 10% test structures. The CoRe optimizer [29, 30] (version 1.1.0) [31] was applied to minimize the loss function. This loss function was equal to that in Reference [30] ($q = 10.9$). The CoRe optimizer hyperparameters were taken from Reference [30], with $\eta_- = 0.55$ and $p_{\mathrm{frozen}} = 0.025$. The weight decay and fraction of frozen weights were set as in Reference [29] for the weights α and β and those associated with the output neuron. Lifelong adaptive data selection [29] was applied during training. Two updates of the weights were carried out during each training

step. The first was based on 1% of the current training energy predictions, and the second one relied on 1% of the training energy and 1% of the training force predictions of the same structures after the first update. In each training step, the selection of structures employed for the update was renewed. To obtain an HDNNP ensemble, 20 individual HDNNPs were trained and the ten best HDNNPs were selected for the ensemble. This selection was based on the sum of the test mean squared errors of energies and atomic force components, whereby the energy error was multiplied by $2500\,\text{Å}^{-2}$.

Active learning considered structures showing uncertainties higher than $3\cdot\text{RMSE}(E_{\text{ML}}^{\text{test}})$, $6\cdot\text{RMSE}(F_{\alpha,n(Q)}^{\text{test}})$, and/or $18\cdot\text{RMSE}(F_{\alpha,n(E')}^{\text{test}})$ for QM-related energies and QM-related atomic force components ($\alpha = x, y, z$) of QM atoms $n(Q)$ and MM atoms within the cutoff sphere of the ML representation $n(E')$, respectively. Structures were disregarded if they included unphysical small interatomic distances. We required a minimum of 20 simulation steps between two structures chosen for active learning. For each simulation, we then selected up to eight structures. The selection was based on maximizing the simulation time separation. 150 AFE simulations were executed in parallel in each active learning iteration. During the first 20 iterations of active learning, only 1000 epochs were used during MLP training to reduce the training time. Afterward, the number of epochs was increased to 5000. The active learning procedure was considered converged when no additional structures were added to the database, or the binding free energy $\Delta G_{\text{bind}}^{\text{ML/MM}}$ did not change within its uncertainties. The uncertainties from the NEQ switching simulations were obtained from sampling the binding free energies from six independent NEQ simulations for each endastate, providing 36 values for $\Delta G_{\text{bind}}^{\text{ML/MM}}$.

Training and prediction of HDNNP ensembles for QM/MM data, as well as active and lifelong learning, was implemented in a development version of the lMLP software [25]. The symmetry functions in Equations (6) and (7) were implemented in an efficient C++ package 'SymmetryFunctions' providing Python and PyTorch bindings for ease of use. All software will be made available on GitHub and is provided on Zenodo[32] or ERDA[25].

S1.4. Alchemical Free Energy Simulations

Absolute binding AFE simulations were performed using Yank 0.25.2 [21] and OpenMM 8.1.1 [18] according to the thermodynamic cycle shown in Figure 2. To prevent the ligand from moving through the simulation box during the protein–ligand complex dissociation simulation, we applied a harmonic restraint between the centers of mass of the protein and ligand with an equilibrium distance of zero. The spring constant was chosen such that the potential reaches $k_{\text{B}}T$ at one radius of gyration of the protein, where k_{B} is the Boltzmann constant and T is the temperature. We applied the following λ-scheme for the protein–ligand complex leg: increase the harmonic protein–ligand restraint from $\lambda = 0$ to 1 in steps of 0.25, annihilate the ligand electrostatic interactions from $\lambda = 1$ to 0 in steps of 0.1, and finally decouple Lennard-Jones interactions between the ligand and environment from $\lambda = 1$ to 0.5 in steps of 0.1 and from $\lambda = 0.5$ to 0 in steps of 0.05. We employed the same λ-scheme without the harmonic restraint step for the solvation of the ligand. We ran one simulation for each λ-state. We utilized the Hamiltonian replica exchange scheme with Gibbs sampling implemented in Yank [33], in which pairs of simulations can swap their alchemical states based on MC moves. The Metropolis criterium was

$$P_{\text{accept}} = \min\left\{1,\ \frac{\exp\{-[u_i(x_j) + u_j(x_i)]\}}{\exp\{-[u_i(x_i) + u_j(x_j)]\}}\right. , \tag{2}$$

where x is the configuration of the system in alchemical state i or j, and u is the reduced potential energy given by the force field of alchemical state i or j.

We propagated the simulation with the default settings in Yank. For the protein–ligand complex, simulations were run for 2×10^4 iterations employing the following protocol: (i) propose Hamiltonian replica exchange MC moves to swap the alchemical states of pairs of simulations, (ii) propose a rigid ligand displacement MC move, (iii) propose a rigid ligand rotation MC move, (iv) reassign Maxwell-Boltzmann velocities at 300 K, (v) 1 ps of Langevin dynamics with a g-BAOAB integrator [34] with a 2 fs time step and $1\,\text{ps}^{-1}$ collision rate. Simulations of the ligand in solution were propagated for 2×10^4 iterations of the same protocol, but without the rigid displacement and rotation MC moves. Simulations were performed at a temperature of 300 K with a MC barostat at 1 bar. The length of bonds to hydrogen atom was constrained. We applied the default settings in Yank for anisotropic dispersion corrections for long-range interactions, whereby the endpoints of each leg in the thermodynamic cycle are reweighted based on energies calculated with an expanded cutoff of $(0.8\,r_{\text{min}})/2$, where r_{min} is the norm of the smallest box vector of the initial system [35]. A similar scheme is employed for the particle-mesh Ewald (PME) summation in Yank, where simulations are run with only the direct space contribution of PME, and endpoints are reweighted to take into account the reciprocal space contribution of PME.

We analyzed the AFE simulations employing Yank. The initial simulation frames were discarded using automated equilibration detection based on the potential energies [36]. To calculate the free energy of binding (Equation (3) of the main text), $\Delta G^{\mathrm{MM}}_{\mathrm{complex}}$, $\Delta G^{\mathrm{MM}}_{\mathrm{solv}}$, and their corresponding respective uncertainties were calculated applying the MBAR [37] as implemented in pymbar [38]. $\Delta G^{\mathrm{MM}}_{\mathrm{complex}}$ also requires a standard state correction to account for the harmonic restraints, which corresponds to releasing the ligand from the harmonic restraint into the volume of a $1\,\mathrm{mol\,L^{-1}}$ solution. The standard state correction was calculated with radially symmetric restraints in OpenMM. The correction is given by

$$\Delta G_{\mathrm{restraint}} = -k_{\mathrm{B}}T\ln(C_0 V_{\mathrm{L}}) \ . \tag{3}$$

Here $C_0 = 0.6022\,\mathrm{nm}^{-3}$ is the standard state concentration, and V_{L} is the volume of the restrained ligand. The latter is obtained by integrating the harmonic potential

$$V_L = \int_0^{r_{\max}} 4\pi r^2 \exp\left[-\frac{1}{2}k(r-r_0)^2\right] \mathrm{d}r \ . \tag{4}$$

The equilibrium distance is denoted by r_0 and $r_{\max}$ is a distance cutoff set to three times the length of the longest simulation box vector.

S1.5. End-State Correction Simulations

Corrections to the end-state free energies associated with going from the MM to the QM/MM representation were determined using bidirectional NEQ switching simulations with the same approach as in References [39] and [40]. We randomly sampled 150 structures (with replacement) from the MD trajectory at the selected thermodynamic state and started a 10 ps forward switching (MM to ML/MM) simulation from each structure. In the forward switching simulations, we interpolated between the MM and ML/MM potential employing

$$E(\lambda) = (1-\lambda)E_{\mathrm{MM}} + \lambda E_{\mathrm{ML/MM}} \ , \tag{5}$$

where λ is a scaling parameter that is updated to $\lambda = n/N$ every 10 time steps. N is the total number and n is the current time step, respectively. Every 10 steps, we calculated the dimensionless work associated with updating the potential for the current structure,

$$\mathrm{work}_t = \frac{1}{k_{\mathrm{B}}T}[E(\lambda_t) - E(\lambda_{t-1})] \ . \tag{6}$$

After completion of the simulation, we calculated the accumulated work of the full switch from MM to ML/MM

$$\mathrm{work}_{\mathrm{MM}\to\mathrm{ML/MM}} = \sum_t \mathrm{work}_t \ . \tag{7}$$

Next, we randomly resampled with replacement 150 structures from the final snapshots of the forward switching simulations, with the probability of sampling a structure X given by

$$w(X) = \exp\left[-\mathrm{work}_{\mathrm{MM}\to\mathrm{ML/MM}}(X)\right] \ , \tag{8}$$

where $\mathrm{work}_{\mathrm{MM}\to\mathrm{ML/MM}}(X)$ is the accumulated work for the forward switching simulation leading to structure X. This approach was chosen to better approximate the equilibrium ensemble of the ML/MM representation. From each resampled structure, we ran a 10 ps equilibration simulation employing ML/MM, followed by a 10 ps switching simulation from the ML/MM to the MM representation. The latter applied the same protocol as the forward switching simulation but with $\lambda = 1 - (n/N)$. From this backward switching simulation, $\mathrm{work}_{\mathrm{ML/MM}\to\mathrm{MM}}$ was obtained. Subsequently, we calculated the correction to the free energy and the associated error applying the Bennett Acceptance Ratio (BAR) between the distributions of $\mathrm{work}_{\mathrm{MM}\to\mathrm{ML/MM}}$ and $-\mathrm{work}_{\mathrm{ML/MM}\to\mathrm{MM}}$ with the pymbar package [38, 41–44]. These corrections are added to the binding free energies as shown in Equation (3) of the main text.

The NEQ switching simulations were run with OpenMM 8.1.2 [18]. We interfaced our ML potential directly to OpenMM using the Python package 'EEForce', which will be available on GitHub.

NEQ switching simulations with ANI-2x were performed using the TorchANI implementation in OpenMM-ML 1.2 [45]. ANI-2x simulations were performed with a 1 fs time step, keeping all hydrogen constraints from the MM system setup. We used the same bidirectional NEQ switching protocol for ANI-2x endstate corrections, with the exception that λ was updated at every time step.

S1.6. Structural analysis

We constructed ML/MM equilibrium ensembles by taking the final structure of each ML/MM equilibration simulation from the NEQ switching cycle. The resampling step and subsequent equilibration simulations should push the distribution of structures toward the equilibrium ML/MM ensemble. We used all six NEQ switching calculations, each with 150 simulations, to construct the ML/MM ensembles. We calculated dihedral angles from these ML/MM ensembles and the endstate simulations from our MM AFE calculations using the MDAnalysis Python package [46]. For NKP1339, we calculated the N^1-N^2-Ru-Cl dihedral angles for both indazoles (indazole 1 to Cl^3 and indazole 2 to Cl^4) and the N^1-N^2-N^2-N^1 dihedral angles between the two indazoles across the Ru center. For 19G, we calculated the C^{AV}-C^{AW}-C^{AQ}-O^{AD}, C^{AW}-C^{AV}-C^{AN}-C^{AL}, C^{AV}-C^{AN}-C^{AL}-C^{AM}, C^{AN}-C^{AL}-C^{AM}-O^{AO}, C^{AL}-C^{AM}-O^{AO}-C^{AT}, and C^{AM}-O^{AO}-C^{AT}-C^{AJ} dihedral angles.

S1.7. Element-Embracing Atom-Centered Symmetry Function Parameters

The cutoff radius $R_c = 5\,\text{Å}$ and the maximum absolute atomic charge $q_{\max} = 1\,e$ were applied for all element-embracing atom-centered symmetry functions (eeACSFs).

Table S1: Parameters of the radial eeACSFs. All combinations of the parameters in each row are applied.

I	h	η
t^Q, t^E	1	4.384960, 7.315969, 12.621850, 23.782024, 54.109124
t^Q	n	3.571065, 5.952354, 10.077045, 18.178648, 37.671588
t^Q	m	4.854966, 8.125365, 14.194768, 27.480122, 66.398697
t^Q	$\overline{n}$	5.374978, 9.039436, 16.026943, 32.018267, 83.174328
t^Q	$\overline{m}$	3.958754, 6.595853, 11.261492, 20.728746, 44.837797

Table S2: Parameters of the angular eeACSFs. All combinations of the parameters in each row are applied.

I	h	γ	λ	ζ	η
$t_j^Q \cdot t_k^Q$	1	1	−1, 1	1, 2, 6	6.919852, 20.696185
$t_j^Q \cdot t_k^Q$	n	1	−1, 1	1, 2, 4	4.687023, 12.059475
$t_j^Q \cdot t_k^Q$	n	−1	−1, 1	1, 2, 4	5.150297, 13.663265
$t_j^Q \cdot t_k^Q$	m	1	−1, 1	1, 3, 7	8.542166, 28.568769
$t_j^Q \cdot t_k^Q$	m	−1	−1, 1	1, 3, 7	7.675911, 24.181530
$t_j^Q \cdot t_k^Q$	$\overline{n}$	1	−1, 1	2, 3, 10	10.701335, 41.587164
$t_j^Q \cdot t_k^Q$	$\overline{n}$	−1	−1, 1	2, 3, 10	9.541125, 34.198192
$t_j^Q \cdot t_k^Q$	$\overline{m}$	1	−1, 1	1, 2, 5	5.670115, 15.575724
$t_j^Q \cdot t_k^Q$	$\overline{m}$	−1	−1, 1	1, 2, 5	6.256063, 17.881461
$t_j^Q \cdot t_k^E + t_j^E \cdot t_k^Q$, $t_j^E \cdot t_k^E$	1		−1, 1	1, 2, 6	4.687023
$t_j^Q \cdot t_k^E + t_j^E \cdot t_k^Q$	n		−1, 1	1, 2, 4	4.687023
$t_j^Q \cdot t_k^E + t_j^E \cdot t_k^Q$	m		−1, 1	1, 3, 7	4.687023
$t_j^Q \cdot t_k^E + t_j^E \cdot t_k^Q$	$\overline{n}$		−1, 1	2, 3, 10	4.687023
$t_j^Q \cdot t_k^E + t_j^E \cdot t_k^Q$	$\overline{m}$		−1, 1	1, 2, 5	4.687023

S1.8. Element Energies

Table S3: Energies of the neutral free atoms in their lowest spin state E_{elem} in Hartree (E_{h}).

Element	E_{elem} / E_{h}
H	−0.498629930
C	−37.751244825
N	−54.466595225
O	−74.914700682
S	−397.800714902
Cl	−459.797166452
Ru	−94.792756987

S2. SUPPORTING RESULTS

S2.1. High Molecular Mechanics Energy Structures

During active learning of the MCL1-19G complex and the solvated 19G ligand, structures were encountered that showed high molecular mechanics (MM) energies ($\sim$ 400 $\mathrm{kJ\,mol^{-1}}$ to 900 $\mathrm{kJ\,mol^{-1}}$ higher than the median). These high energies are caused by short bond distances that MM force fields cannot accurately describe. Figure S1 shows an example of such a structure. This structure has unusually short O–H and C–H bonds between atoms that are not covalently bonded according to the MM topology. Such a transition state-like conformation can be handled by quantum mechanics but not by molecular mechanics.

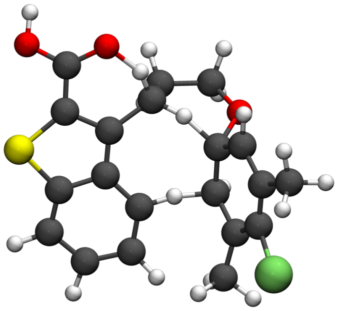

Figure S1: Structure from active learning showing a very high MM energy and unusually short C-H-C and O-H-C bonds.

S2.2. Endstate corrections with ANI-2x

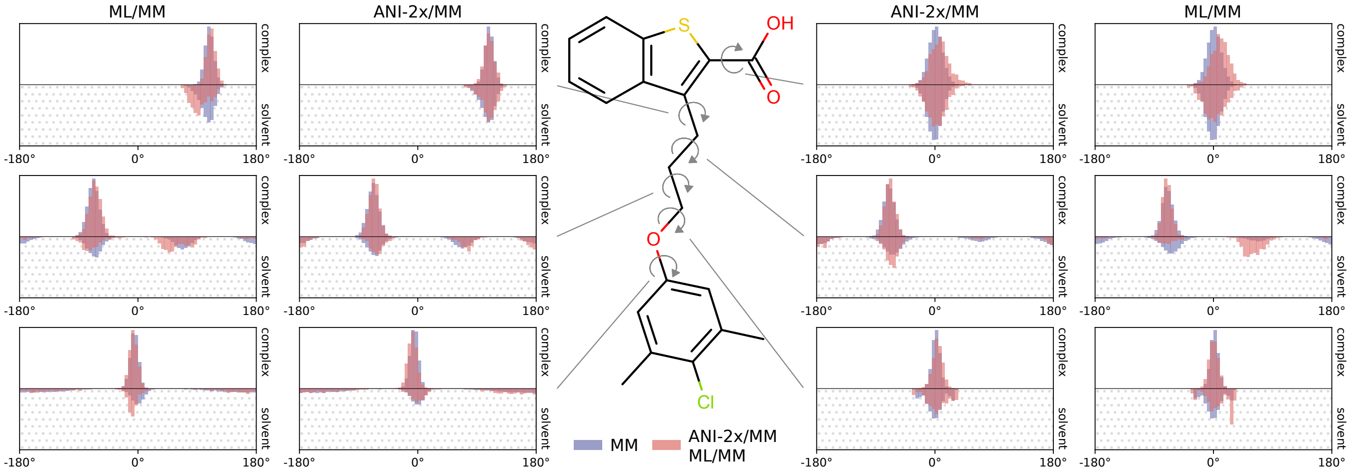

Figure S2: Comparison of dihedral angle distributions produced by our MLP and ANI-2x for 19G in the MCL1-19G complex and in solution. Distributions were calculated from simulations with the MM potential (blue) and ANI-2x/MM or our own ML/MM potential (red). The columns of plots are labeled according to the force field. ANI-2x/MM and ML/MM distributions were calculated over parallel NEQ switching simulations, using the final structure of the equilibration step.

S2.3. Structural Analysis for NKP1339

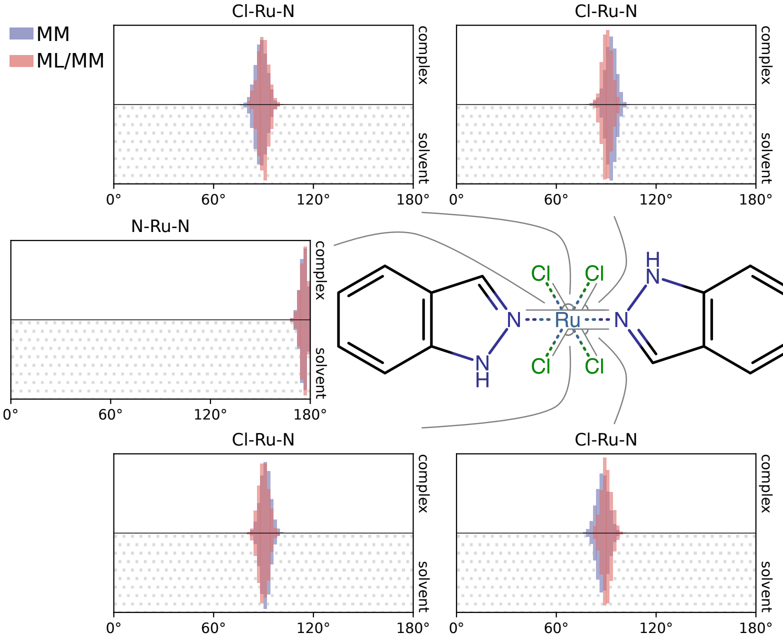

Figure S3: Angle distributions in the Ru center for NKP1339 in the GRP78-NKP1339 complex and in solution. Distributions were calculated from simulations with the MM potential (blue) and ML/MM potential (red). ML/MM distributions were calculated over parallel NEQ switching simulations, using the final structure of the ML/MM equilibration step.

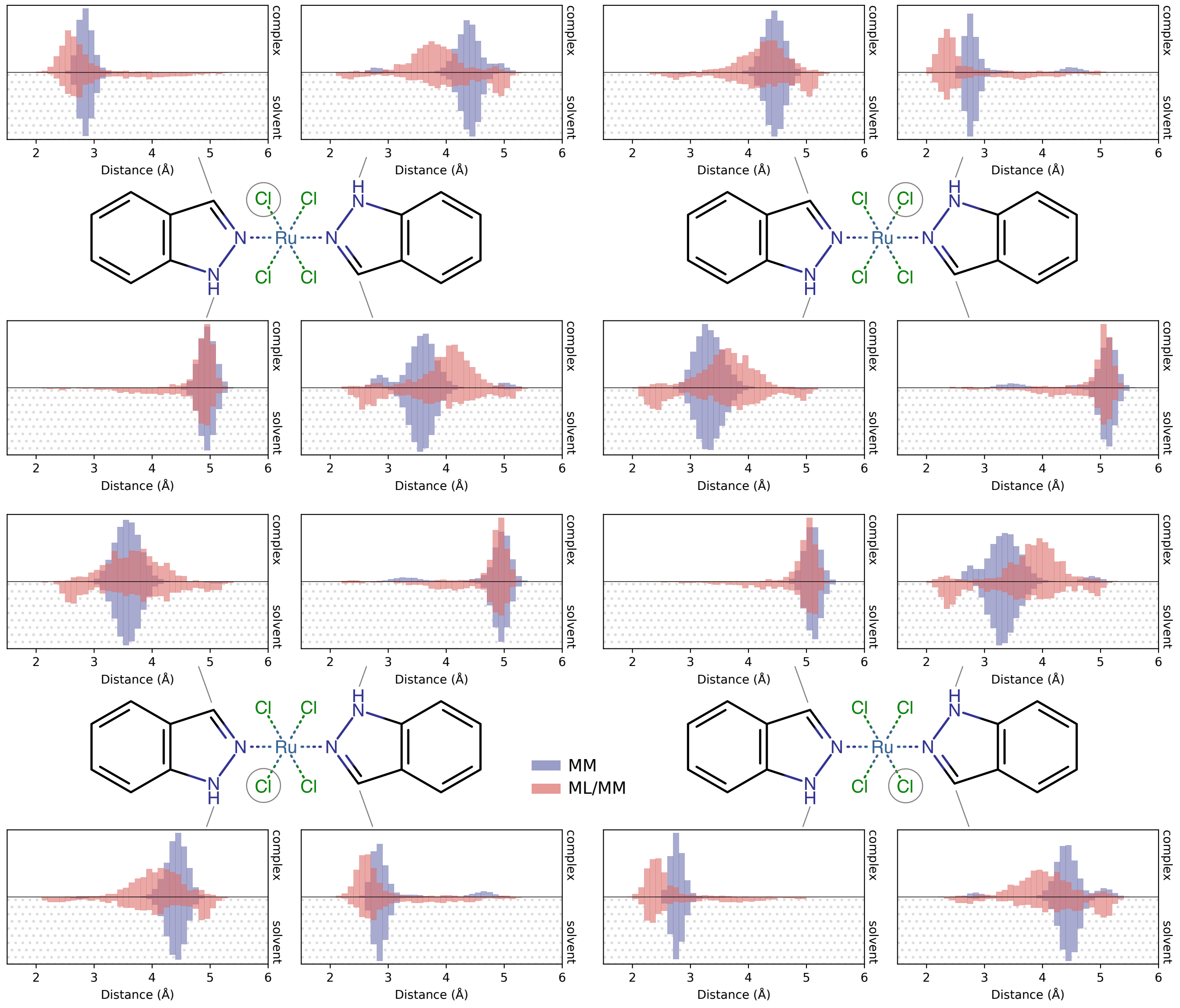

Figure S4: Cl-H distance distributions for NKP1339 in the GRP78-NKP1339 complex and in solution. Each set of four plots corresponds to the circled Cl. Distributions were calculated from simulations with the MM potential (blue) and ML/MM potential (red). ML/MM distributions were calculated over parallel NEQ switching simulations, using the final structure of the ML/MM equilibration step.

S3. COMPUTATIONAL FRAMEWORK

To avoid any time bottlenecks that may arise due to the high computational cost of QM/MM calculations, we exploited a distributed computing framework, as shown in Figure S5. This framework employs a central database accessible from multiple high-performance computing clusters. The database is based on the SCINE database framework [28]. The database contains the molecular structures, properties for the structures, such as QM/MM energies, forces, charges, and force field information, and the calculation input and output for the QM/MM calculations. Initially, the database is populated with the structures and calculation inputs based on the end state trajectories of the classical MM alchemical free energy calculations. Then the QM/MM calculations are executed through multiple instances of the program Puffin [47], which acts as a client for the database. Puffin searches the database for calculations not already completed or running, performs the QM/MM calculation through an interface to SCINE SWOOSE [6, 9, 25] and Turbomole [26, 27], and returns the results to the database.

Once the QM/MM calculations are completed, the machine learning potential is trained with the program lMLP [25] by reading structures, forces, and energies from the database. Afterward, the NEQ simulations are run with the

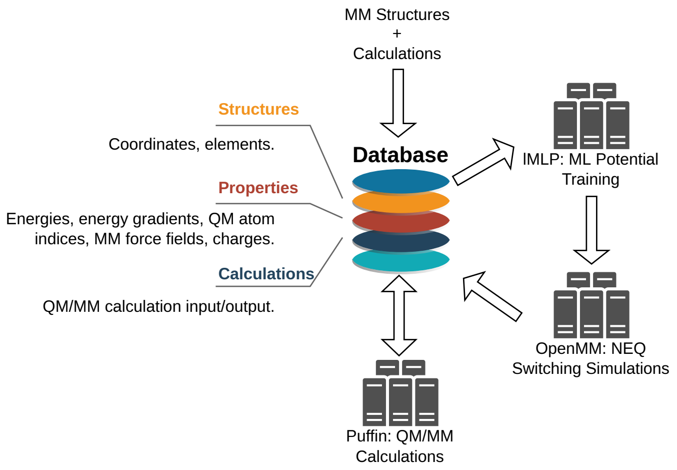

Figure S5: Sketch of the computational framework used to exploit a central database and distribute calculations.

machine learning potential, and the structures considered for active learning are written to the database, including a new calculation entry signaling Puffin to perform the QM/MM calculations. Since multiple computing centers can access the database, this framework allows us to easily scale the number of Puffins and parallelize the execution of the QM/MM calculations.

[1] K. Lindorff-Larsen, S. Piana, K. Palmo, P. Maragakis, J. L. Klepeis, R. O. Dror, and D. E. Shaw, Improved side-chain torsion potentials for the Amber ff99SB protein force field, Proteins: Structure, Function, and Bioinformatics **78**, 1950 (2010).

[2] J. Wang, R. M. Wolf, J. W. Caldwell, P. A. Kollman, and D. A. Case, Development and Testing of a General Amber Force Field, J. Comput. Chem. **25**, 1157 (2004).

[3] J. Wang, R. M. Wolf, J. W. Caldwell, P. A. Kollman, and D. A. Case, Erratum: "Development and testing of a general amber force field", J. Comput. Chem. **26**, 114 (2005).

[4] D. L. Mobley, C. C. Bannan, A. Rizzi, C. I. Bayly, J. D. Chodera, V. T. Lim, N. M. Lim, K. A. Beauchamp, D. R. Slochower, M. R. Shirts, M. K. Gilson, and P. K. Eastman, Escaping Atom Types in Force Fields Using Direct Chemical Perception, J. Chem. Theory Comput. **14**, 6076 (2018).

[5] W. L. Jorgensen, J. Chandrasekhar, J. D. Madura, R. W. Impey, and M. L. Klein, Comparison of simple potential functions for simulating liquid water, J. Chem. Phys. **79**, 926 (1983).

[6] C. Brunken and M. Reiher, Self-Parametrizing System-Focused Atomistic Models, J. Chem. Theory Comput. **16**, 1646 (2020).

[7] C. Brunken and M. Reiher, Automated Construction of Quantum–Classical Hybrid Models, J. Chem. Theory Comput. **17**, 3797 (2021).

[8] C. Brunken, K. Csizi, M. Steiner, T. Weymuth, and M. Reiher, qcscine/swoose: Release 2.0.0 (Version 2.0.0), Zenodo 10.5281/zenodo.11551858 (2024).

[9] T. Weymuth, J. P. Unsleber, P. L. Türtscher, M. Steiner, J.-G. Sobez, C. H. Müller, M. Mörchen, V. Klasovita, S. A. Grimmel, M. Eckhoff, K.-S. Csizi, F. Bosia, M. Bensberg, and M. Reiher, SCINE—Software for chemical interaction networks, J. Chem. Phys. **160**, 222501 (2024).

[10] C. Adamo and V. Barone, Toward reliable density functional methods without adjustable parameters: The PBE0 model, J. Chem. Phys. **110**, 6158 (1999).

[11] S. Grimme, J. Antony, S. Ehrlich, and H. Krieg, A consistent and accurate ab initio parametrization of density functional dispersion correction (DFT-D) for the 94 elements H-Pu, J. Chem. Phys. **132**, 154104 (2010).

[12] S. Grimme, S. Ehrlich, and L. Goerigk, Effect of the damping function in dispersion corrected density functional theory, J. Comput. Chem. **32**, 1456 (2011).

[13] F. Weigend and R. Ahlrichs, Balanced basis sets of split valence, triple zeta valence and quadruple zeta valence quality for h to rn: Design and assessment of accuracy, Phys. Chem. Chem. Phys. **7**, 3297 (2005).

[14] A. Friberg, D. Vigil, B. Zhao, R. N. Daniels, J. P. Burke, P. M. Garcia-Barrantes, D. Camper, B. A. Chauder, T. Lee, E. T. Olejniczak, and S. W. Fesik, Discovery of potent myeloid cell leukemia 1 (Mcl-1) inhibitors using fragment-based methods and structure-based design, J. Med. Chem. **56**, 15 (2013).

[15] H. M. Berman, J. Westbrook, Z. Feng, G. Gilliland, T. N. Bhat, H. Weissig, I. N. Shindyalov, and P. E. Bourne, The protein data bank, Nucleic Acids Res. **28**, 235 (2000).

[16] A. T. Macias, D. S. Williamson, N. Allen, J. Borgognoni, A. Clay, Z. Daniels, P. Dokurno, M. J. Drysdale, G. L. Francis, C. J. Graham, R. Howes, N. Matassova, J. B. Murray, R. Parsons, T. Shaw, A. E. Surgenor, L. Terry, Y. Wang, M. Wood, and A. J. Massey, Adenosine-Derived Inhibitors of 78 kDa Glucose Regulated Protein (Grp78) ATPase: Insights into Isoform Selectivity, J. Med. Chem. **54**, 4034 (2011).

[17] J. A. Izaguirre, C. R. Sweet, and V. S. Pande, Multiscale Dynamics of Macromolecules Using Normal Mode Langevin, Pac. Symp. Biocomput. , 240 (2010).

[18] P. Eastman, R. Galvelis, R. P. Peláez, C. R. A. Abreu, S. E. Farr, E. Gallicchio, A. Gorenko, M. M. Henry, F. Hu, J. Huang, A. Krämer, J. Michel, J. A. Mitchell, V. S. Pande, J. P. Rodrigues, J. Rodriguez-Guerra, A. C. Simmonett, S. Singh, J. Swails, P. Turner, Y. Wang, I. Zhang, J. D. Chodera, G. De Fabritiis, and T. E. Markland, OpenMM 8: Molecular Dynamics Simulation with Machine Learning Potentials, J. Phys. Chem. B **128**, 109 (2024).

[19] K.-H. Chow and D. M. Ferguson, Isothermal-isobaric molecular dynamics simulations with Monte Carlo volume sampling, Comput. Phys. Commun. **91**, 283 (1995).

[20] J. Åqvist, P. Wennerström, M. Nervall, S. Bjelic, and B. O. Brandsdal, Molecular dynamics simulations of water and biomolecules with a Monte Carlo constant pressure algorithm, Chem. Phys. Lett. **384**, 288 (2004).

[21] A. Rizzi, J. Chodera, L. Naden, K. Beauchamp, S. Albanese, P. Grinaway, D. Prada-Gracia, B. Rustenburg, Ajsilveira, S. Saladi, K. Boehm, J. Gmach, and J. Rodríguez-Guerra, choderalab/yank: 0.25.2 - Bugfix release (2019), DOI: 10.5281/ZENODO.3534289.

[22] E. Bitzek, P. Koskinen, F. Gähler, M. Moseler, and P. Gumbsch, Structural relaxation made simple, Phys. Rev. Lett. **97**, 170201 (2006).

[23] J. P. Perdew, K. Burke, and M. Ernzerhof, Generalized Gradient Approximation Made Simple, Phys. Rev. Lett. **77**, 3865 (1996).

[24] F. Weigend and R. Ahlrichs, Balanced basis sets of split valence, triple zeta valence and quadruple zeta valence quality for H to Rn: Design and assessment of accuracy, Phys. Chem. Chem. Phys. **7**, 3297 (2005).

[25] M. Bensberg, M. Eckhoff, F. E. Thomasen, M. S. Teynor, W. Bro-Jørgensen, V. Sora, T. Weymuth, R. T. Husistein, F. E. Knudsen, A. Krogh, K. Lindorff-Larsen, M. Reiher, and G. C. Solomon, Supplemental Material: Machine Learning Enhanced Calculation of Quantum-Classical Binding Free Energies (2025), https://sid.erda.dk/cgi-sid/ls.py?share_id=L6JhSY0c1P (this link will be frozen and a DOI will be generated after completing the peer review

process.).
[26] R. Ahlrichs, M. Bär, M. Häser, H. Horn, and C. Kölmel, Electronic structure calculations on workstation computers: The program system turbomole, Chem. Phys. Lett. **162**, 165 (1989).
[27] TURBOMOLE V7.4.1 2019, a development of University of Karlsruhe and Forschungszentrum Karlsruhe GmbH, 1989-2007, TURBOMOLE GmbH, since 2007; available from `http://www.turbomole.com`. (2019).
[28] M. Bensberg, S. A. Grimmel, J.-G. Sobez, M. Steiner, P. L. Türtscher, J. P. Unsleber, and M. Reiher, qcscine/database: Release 1.3.0 (2023), DOI: 10.5281/zenodo.6695495.
[29] M. Eckhoff and M. Reiher, Lifelong machine learning potentials, J. Chem. Theory Comput. **19**, 3509 (2023).
[30] M. Eckhoff and M. Reiher, Core optimizer: an all-in-one solution for machine learning, Mach. Learn.: Sci. Technol. **5**, 015018 (2024).
[31] M. Eckhoff and M. Reiher, ReiherGroup/CoRe_optimizer: Release 1.1.0, Zenodo 10.5281/zenodo.11551858 (2024).
[32] M. Bensberg, M. Eckhoff, R. T. Husistein, M. S. Teynor, W. Bro-Jørgensen, F. E. Thomasen, V. Sora, A. Krogh, K. Lindorff-Larsen, G. C. Solomon, T. Weymuth, and M. Reiher, Supplemental Material: Hierarchical quantum embedding by machine learning for large molecular assemblies (2025), DOI: 10.5281/zenodo.14971120.
[33] J. D. Chodera and M. R. Shirts, Replica exchange and expanded ensemble simulations as Gibbs sampling: Simple improvements for enhanced mixing, J. Chem. Phys. **135**, 194110 (2011).
[34] B. Leimkuhler and C. Matthews, Efficient molecular dynamics using geodesic integration and solvent–solute splitting, Proc. R. Soc. A **472**, 20160138 (2016).
[35] M. R. Shirts, D. L. Mobley, J. D. Chodera, and V. S. Pande, Accurate and Efficient Corrections for Missing Dispersion Interactions in Molecular Simulations, J. Phys. Chem. B **111**, 13052 (2007).
[36] J. D. Chodera, A Simple Method for Automated Equilibration Detection in Molecular Simulations, J. Chem. Theory Comput. **12**, 1799 (2016).
[37] M. R. Shirts and J. D. Chodera, Statistically optimal analysis of samples from multiple equilibrium states, J. Chem. Phys. **129**, 124105 (2008).
[38] M. Shirts, K. Beauchamp, L. Naden, J. Chodera, jaimergp, S. Martiniani, C. Stern, M. Henry, M. Thompson, J. Fass, R. Gowers, R. T. McGibbon, B. Dice, C. Jones, D. L. Dotson, F. York, I. Pulido, and T. Burgin, choderalab/pymbar: 4.0.3 Support for Python 3.12 and Jax 0.3.25+ (2024), DOI: 10.5281/ZENODO.10849928.
[39] D. A. Rufa, H. E. Bruce Macdonald, J. Fass, M. Wieder, P. B. Grinaway, A. E. Roitberg, O. Isayev, and J. D. Chodera, Towards chemical accuracy for alchemical free energy calculations with hybrid physics-based machine learning / molecular mechanics potentials, bioRxiv 10.1101/2020.07.29.227959 (2020).
[40] S. Tkaczyk, J. Karwounopoulos, A. Schöller, H. L. Woodcock, T. Langer, S. Boresch, and M. Wieder, Reweighting from Molecular Mechanics Force Fields to the ANI-2x Neural Network Potential, J. Chem. Theory Comput. **20**, 2719 (2024).
[41] C. H. Bennett, Efficient estimation of free energy differences from Monte Carlo data, J. Comput. Phys. **22**, 245 (1976).
[42] C. Jarzynski, Nonequilibrium Equality for Free Energy Differences, Phys. Rev. Lett. **78**, 2690 (1997).
[43] G. E. Crooks, Path-ensemble averages in systems driven far from equilibrium, Phys. Rev. E **61**, 2361 (2000).
[44] M. R. Shirts, E. Bair, G. Hooker, and V. S. Pande, Equilibrium Free Energies from Nonequilibrium Measurements Using Maximum-Likelihood Methods, Phys. Rev. Lett. **91**, 140601 (2003).
[45] X. Gao, F. Ramezanghorbani, O. Isayev, J. S. Smith, and A. E. Roitberg, TorchANI: A Free and Open Source PyTorch-Based Deep Learning Implementation of the ANI Neural Network Potentials, J. Chem. Inf. Model. **60**, 3408 (2020).
[46] N. Michaud-Agrawal, E. J. Denning, T. B. Woolf, and O. Beckstein, MDAnalysis: A toolkit for the analysis of molecular dynamics simulations, J. Comput. Chem. **32**, 2319 (2011).
[47] M. Bensberg, C. Brunken, K.-S. Csizi, S. Grimmel, S. Gugler, J.-G. Sobez, M. Steiner, P. L. Türtscher, J. P. Unsleber, T. Weymuth, and M. Reiher, qcscine/puffin: Release 2.0.0 (2024), dOI: 10.5281/zenodo.13372960.
